# Photothermal Catalyst Engineering: Hydrogenation of Gaseous CO_2_ with High Activity and Tailored Selectivity

**DOI:** 10.1002/advs.201700252

**Published:** 2017-07-25

**Authors:** Jia Jia, Hong Wang, Zhuole Lu, Paul G. O'Brien, Mireille Ghoussoub, Paul Duchesne, Ziqi Zheng, Peicheng Li, Qiao Qiao, Lu Wang, Alan Gu, Abdinoor A. Jelle, Yuchan Dong, Qiang Wang, Kulbir Kaur Ghuman, Thomas Wood, Chenxi Qian, Yue Shao, Chenyue Qiu, Miaomiao Ye, Yimei Zhu, Zheng‐Hong Lu, Peng Zhang, Amr S. Helmy, Chandra Veer Singh, Nazir P. Kherani, Doug D. Perovic, Geoffrey A. Ozin

**Affiliations:** ^1^ Department of Materials Science and Engineering University of Toronto 184 College Street Toronto Ontario M5S 3E4 Canada; ^2^ Materials Chemistry and Nanochemistry Research Group Solar Fuels Cluster Department of Chemistry University of Toronto 80 St. George Street Toronto Ontario M5S 3H6 Canada; ^3^ Department of Chemical Engineering and Applied Chemistry University of Toronto 200 College Street Toronto Ontario M5S 3E5 Canada; ^4^ Department of Mechanical Engineering Lassonde School of Engineering York University Toronto M3J 1P3 Canada; ^5^ Department of Chemistry Dalhousie University 6274 Coburg Road, P.O. Box 15000 Halifax Nova Scotia B3H 4R2 Canada; ^6^ Condensed Matter Physics and Materials Science Department Brookhaven National Laboratory Upton NY 11973 USA; ^7^ Department of Physics Temple University Philadelphia PA 19122 USA; ^8^ State Key Laboratory of Coal Conversion Institute of Coal Chemistry The Chinese Academy of Sciences Taiyuan 030001 P. R. China; ^9^ Zhejiang Key Laboratory of Drinking Water Safety and Distribution Technology Zhejiang University Hangzhou 310058 P. R. China; ^10^ Department of Electrical and Computing Engineering University of Toronto 10 King's College Road Toronto Ontario M5S 3G4 Canada

**Keywords:** CO_2_ conversion, photothermal catalysts, size effects, tunable selectivity

## Abstract

This study has designed and implemented a library of hetero‐nanostructured catalysts, denoted as Pd@Nb_2_O_5_, comprised of size‐controlled Pd nanocrystals interfaced with Nb_2_O_5_ nanorods. This study also demonstrates that the catalytic activity and selectivity of CO_2_ reduction to CO and CH_4_ products can be systematically tailored by varying the size of the Pd nanocrystals supported on the Nb_2_O_5_ nanorods. Using large Pd nanocrystals, this study achieves CO and CH_4_ production rates as high as 0.75 and 0.11 mol h^−1^ g_Pd_^−1^, respectively. By contrast, using small Pd nanocrystals, a CO production rate surpassing 18.8 mol h^−1^ g_Pd_^−1^ is observed with 99.5% CO selectivity. These performance metrics establish a new milestone in the champion league of catalytic nanomaterials that can enable solar‐powered gas‐phase heterogeneous CO_2_ reduction. The remarkable control over the catalytic performance of Pd@Nb_2_O_5_ is demonstrated to stem from a combination of photothermal, electronic and size effects, which is rationally tunable through nanochemistry.

## 1 Introduction

The energy crisis and the worsening global climate, caused by the consistent utilization of fossil fuels, have triggered enormous research attention directed at converting CO_2_ into value‐added chemicals and fuels.^1^ A key challenge is the development of highly active and selective photocatalysts capable of reducing carbon dioxide using renewable solar energy and a source of hydrogen. Among different approaches, gas‐phase photoreduction of CO_2_ is considered as a promising strategy, benefiting from its potential to be easily integrated into existing chemical and petrochemical industry infrastructure.^2^ Reduction to practice, however, has hitherto been hindered by low conversion rates, poor selectivity, and limited stability.

Powered by both the heat and light from the sun, the photothermal strategy is extremely appealing for developing superior gas‐phase CO_2_ reduction catalysts.^3^ The photothermal effect can be attributed to different mechanisms. In this context, metal–metal oxide hetero‐nanostructures are a class of materials with optical properties that make them compelling choices as photothermal CO_2_ reduction catalysts. The photothermal effect in these hetero‐nanostructure materials arises from broadband optical absorption and nonradiative relaxation of photoexcited surface plasmons or intra‐/interband electrons in the metal nanocomponents.^4^

Several groups have demonstrated that surface electronic properties can greatly influence photocatalytic CO_2_ reduction rates and selectivity in metal–metal oxide hetero‐nanostructures. For example, charge transfer between the metal and metal oxide constituents can influence the surface charge on the metal nanocrystal and hence its activity and selectivity toward CO_2_ reduction.^5^ Another effective approach to improve the photocatalytic performance for CO_2_ reduction is to engineer size, shape, or a dynamic restructuring of the nanocrystals.^6^

Herein, we combine these strategies to tune and optimize the photothermal CO_2_ hydrogenation activity and selectivity of nanostructured Pd@Nb_2_O_5_ toward either CO or CH_4_ reduction products. A library of Pd@Nb_2_O_5_ nanomaterials with gradually increasing Pd nanocrystal sizes and loadings is synthesized and investigated as photothermal catalysts for the reduction of CO_2_. In a batch reactor under simulated light with illumination intensities of 4.2 W cm^−2^, using 0.1% Pd@Nb_2_O_5_, a CO production rate as high as 18.8 mol h^−1^ g_Pd_^−1^ is observed with 99.5% CO selectivity. In stark contrast, using 10% Pd@Nb_2_O_5_, we achieve a CO production rate surpassing 0.75 mol h^−1^ g_Pd_^−1^ and a CH_4_ production rate of 0.11 mol h^−1^ g_Pd_^−1^. The selectivity to methane on 10% Pd@Nb_2_O_5_ is higher by a factor of 24 than that on 0.1% Pd@Nb_2_O_5_. To simulate more industrially relevant conditions, photocatalytic measurements were also carried out in a gas‐phase flow‐photoreactor using an illumination intensity of 2.1 W cm^−2^. A remarkable light‐driven CO_2_‐to‐CO conversion rate of 0.91 mol h^−1^ g_Pd_^−1^ was obtained with an unprecedented turnover frequency (TOF) as high as 0.61 s^−1^ and an energy conversion efficiency exceeding 0.23%. This represents a new milestone in the champion league of gas‐phase light powered CO_2_ reduction catalysts in the absence of external heating. The results of this study show that “photothermal engineering” of the catalytic properties of Pd@Nb_2_O_5_ hetero‐nanostructures can be used to tailor the activity and selectivity of the gas‐phase CO_2_ reduction reaction, an advance that with further materials and process engineering could enable the development of CO_2_ refineries powered by the heat and light of the sun.

## 2 Results

### 2.1 Synthesis and Characterization of Pd@Nb_2_O_5_ Hetero‐Nanostructures

**Figure 1:**
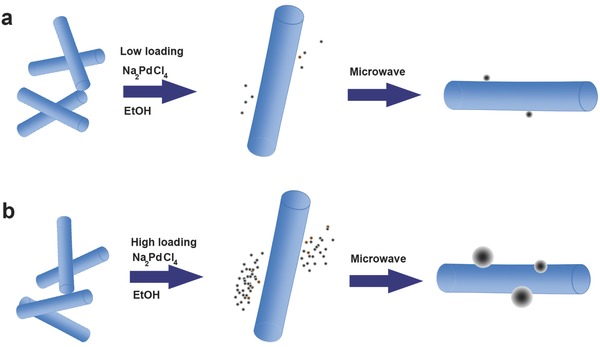
Schematic illustration of the growth of Pd nanocrystals on Nb_2_O_5_ nanorods: a) low loading of the Pd precursor and b) high loading of the Pd precursor.

A library of Pd@Nb_2_O_5_ samples with gradually increasing Pd loadings, labeled as 0.1% to 15% Pd (which is associated with the wt% of Pd in the samples), were synthesized via a two‐step process (**Figure** **1**). The detailed synthetic procedure is provided in the “Experimental Section.” Briefly, nanocrystalline Nb_2_O_5_ nanorods with lengths ranging from less than a hundred nanometers to a few hundred nanometers were synthesized through a facile hydrothermal reaction (Figure S1, Supporting Information). Pd@Nb_2_O_5_ samples were prepared via a straightforward microwave‐assisted hydrothermal reduction strategy using Na_2_PdCl_4_. The elemental Pd content in each Pd@Nb_2_O_5_ sample was determined by inductively coupled plasma‐atomic emission spectroscopy (ICP‐AES), with the results being listed in Table S1 (Supporting Information).

**Figure 2:**
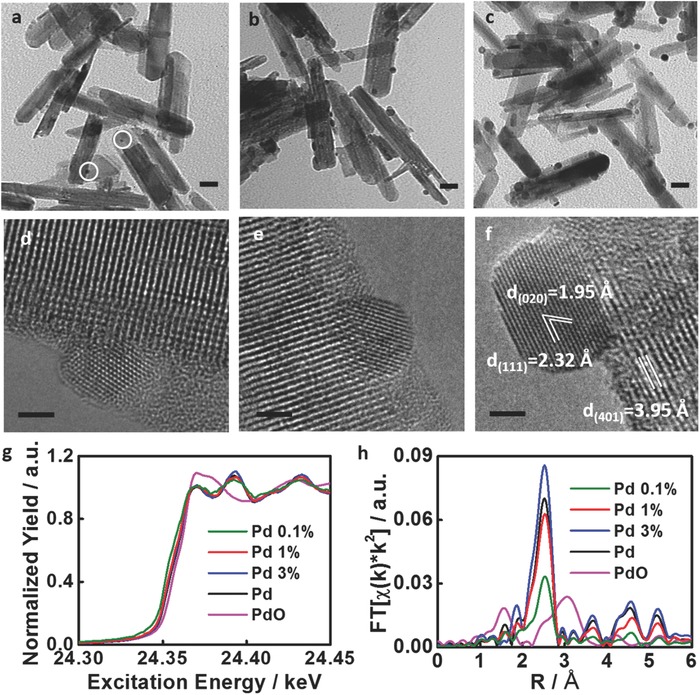
Morphology and structural analysis of Pd@Nb_2_O_5_ samples. Bright field TEM images of a) 0.1% Pd@Nb_2_O_5_ sample; b) 3% Pd@Nb_2_O_5_ sample; and c) 10% Pd@Nb_2_O_5_ sample; scale bar: 20 nm. HRTEM image of d) 0.1% Pd@Nb_2_O_5_ sample; e) 3% Pd@Nb_2_O_5_ sample; and f) 10% Pd@Nb_2_O_5_ sample; scale bar: 2 nm. g) Pd K‐edge XANES spectra from the supported Pd nanocrystals. h) Pd K‐edge FT‐EXAFS spectra from the supported Pd nanocrystals.

**Figure** **2**a–c depicts the transmission electron microscopy (TEM) images of Pd@Nb_2_O_5_ samples with loadings of 0.1%, 3%, and 10%. High‐resolution TEM (HRTEM) images (Figure 2d–f) show the heterojunction structure of Pd nanocrystals interfaced with Nb_2_O_5_ nanorods, in which the atomic number difference and associated mass‐thickness phase contrast provide very clear images of small Pd nanocrystals situated on top of Nb_2_O_5_ nanorods. As revealed by the TEM images (Figure 2; Figure S1, Supporting Information) and a size distribution study shown in Figure S2 (Supporting Information), the population of Pd nanocrystals monotonically increases with Pd loading. The mean diameter of the Pd nanocrystals grows from 2–3 nm for 0.1% Pd@Nb_2_O_5_ to 5–15 nm for 10% Pd@Nb_2_O_5_. A close observation of these Pd@Nb_2_O_5_ samples (Figure 2d,e; Figure S3, Supporting Information) indicates that for 0.1% Pd@Nb_2_O_5_ and 3% Pd@Nb_2_O_5_, the smaller Pd nanocrystals present round‐shaped morphologies without any obvious surface facets. By contrast, clearly defined facets are observed for the larger Pd nanocrystals seen in Figure 2f, where the nanocrystal is viewed along its [$\bar{1}$01] direction. According to Figure 2f and Figure S3a (Supporting Information), the lattice constant along the nanorod axis was determined to be 3.95 Å, in agreement with the lattice spacing of the (401) plane of Nb_2_O_5_, implying that the growth of the Nb_2_O_5_ nanorods proceeds along the [010] habit direction. The observed *d*‐spacings of the Pd (111) and Pd (020) planes are measured to be 2.32 and 1.95 Å, respectively, and the HRTEM image revealed the preferential growth of (111) facets in the Pd nanocrystal. The observed interfacial orientation relationship between the Pd and Nb_2_O_5_ defines that Pd (111) is aligned parallel to the (401) plane of Nb_2_O_5_.

The composition and phase of the Pd nanocrystals were further characterized by powder X‐ray diffraction (PXRD) and synchrotron extended X‐ray absorption fine structure (EXAFS) spectroscopy. As shown in Figure S4 (Supporting Information), the characteristic PXRD patterns of metallic Pd were observed for Pd@Nb_2_O_5_ samples with Pd loading higher than 5%. For low loading samples with Pd nanocrystals in the size range of 5 nm or lower, this is more difficult to observe because of X‐ray line broadening. To provide more insight into the smallest Pd nanocrystals, synchrotron radiation EXAFS spectroscopy was utilized to unravel the phase and atomic structure of the Pd@Nb_2_O_5_ samples with low loadings. The obtained spectra for these Pd@Nb_2_O_5_ samples (Figure 2g) were clearly indicative of metallic Pd bonding environments, with no significant contributions from oxidized species such as PdO. Additionally, the decreasing oscillation intensities in the early EXAFS region (beyond *≈*24.37 keV) reflect a decrease in the coordination number (CN) of Pd atoms for these samples relative to the bulk Pd reference material (Table S2, Supporting Information). These results suggest that the size of the supported Pd nanoparticles (NPs) is reduced in samples at low Pd loadings. Qualitative analysis of the Pd K‐edge EXAFS spectra from the supported Pd nanoparticles, as shown in Figure 2h, revealed a trend toward decreasing intensity of the Pd–Pd scattering peak in the region of 2.5–3.0 Å as the Pd loading was decreased. Because these peak intensities correlate with CN, this observation further supports the conclusion that lower loadings of Pd result in the formation of smaller nanoparticles. As for the very low CN for the 0.1% Pd nanoparticle sample, the obtained value of CN = 6.4 suggests that the average nanoparticle may be as small as 10–20 atoms, based on previous (analogous) studies of metal‐supported Pt nanoclusters.^7^ This conclusion seems reasonable for given literature evidence supporting the exceptional stability of Pt_13_ clusters (CN ≈ 6).^8^

**Figure 3:**
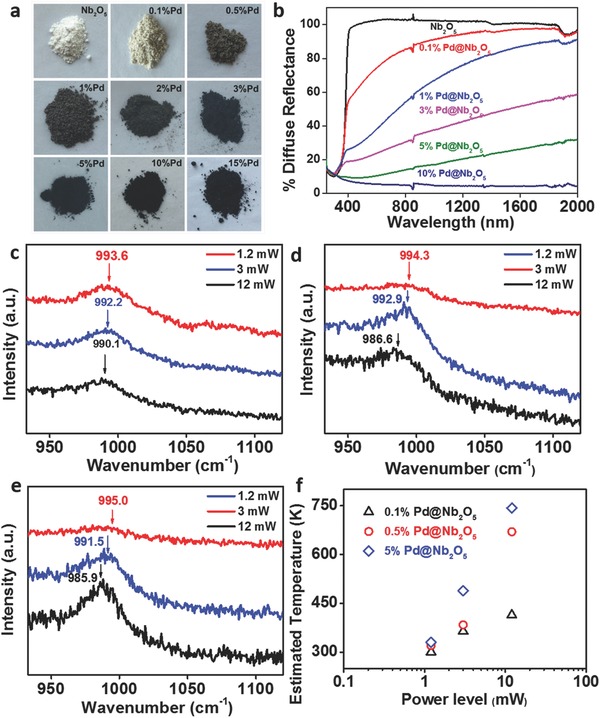
Optical and Raman characterization of Pd@Nb_2_O_5_ samples. a) Digital photographs of pristine Nb_2_O_5_ and Pd@Nb_2_O_5_ samples with different loadings of Pd. b) Diffuse reflectance spectra of pristine Nb_2_O_5_ and different Pd@Nb_2_O_5_ samples dispersed on borosilicate filter films. Dependence of the Raman frequency for νNb=O stretching vibrations of c) 0.1% Pd@Nb_2_O_5,_ d) 0.5% Pd@Nb_2_O_5_, and e) 5% Pd@Nb_2_O_5_ at different incident light power levels. f) The estimated temperatures of 0.1%, 0.5%, and 5% Pd loaded onto Nb_2_O_5_ nanorods at different incient light power levels.

Pristine Nb_2_O_5_ exhibits semiconducting behavior with a strong electronic bandgap absorption only in the ultraviolet (UV) spectral range with a wavelength less than 400 nm (**Figure** **3**b, black curve). The color of Pd@Nb_2_O_5_ hetero‐nanostructures varies from gray to black with increasing loadings of Pd (Figure 3a). Accordingly, the Pd@Nb_2_O_5_ nanostructures efficiently absorb broadband light covering the full spectral range (250–2000 nm), with a monotonic decrease in diffuse reflectance with the increase of Pd loading. According to Mie theory,^9^ the surface plasmon resonance absorption of small Pd nanocrystals (size less than 10 nm) locates at 200–250 nm in the UV wavelength range,^9^ while the visible and near infrared (NIR) light absorption of Pd nanocrystals is dominated by the interband electron transitions (between the d band and s–p conduction band) and intraband transitions (between filled and empty states in the d and s–p bands).^4^, ^10^ Therefore, the noticeable optical absorption in the visible and near‐infrared wavelength range of Pd@Nb_2_O_5_ hetero‐nanostructures, which grows with Pd loading, primarily originates from interband and intraband transitions of Pd nanocrystals.

The local temperature of the hetero‐nanostructures under solar illumination is the key to photothermal catalysis. To appraise the photothermal effect arising from different Pd loadings on the temperature of the nanostructure, Raman measurements were carried out on pristine Nb_2_O_5_ and Pd@Nb_2_O_5_ hetero‐nanostructures to directly measure the local temperatures of the samples. The local temperature was extracted from an analysis of the observed linear shift in the position of the niobium—oxygen phonon modes with increasing temperature.^11^ Figure 3c–e shows the νNb=O stretching modes for 0.1% Pd@Nb_2_O_5_, 0.5% Pd@Nb_2_O_5_, and 5% Pd@Nb_2_O_5_ at around 990 cm^−1^ under a set of different incident laser power levels. At the power level of 12 mW, the local temperatures of Pd@Nb_2_O_5_ samples are estimated to be 414 K for 0.1% Pd@Nb_2_O_5_ and 670 K for 0.5% Pd@Nb_2_O_5_ while the temperature of 5% Pd@Nb_2_O_5_ at the same power level is estimated to be as high as 743 K (Figure 3f). This trend clearly depicts an enhancement of the photothermal effect with loading and size of the Pd “nanoheaters” decorated on the Nb_2_O_5_ nanorods. Although the laser excitation utilized in the Raman measurement is different from the irradiation from the Xe lamp in the photothermal catalytic testing, the Raman measurements allow one to qualitatively compare relative light‐to‐heat conversion capabilities between different Pd@Nb_2_O_5_ samples. Together, the optical and Raman measurements demonstrate that the broadband optical absorption of Pd@Nb_2_O_5_ provides the underlying physicochemical driving force for the significant photothermal effect observed for nanostructured Pd@Nb_2_O_5_.

### 2.2 Photothermal Materials Engineering of the Gas‐Phase CO_2_ Catalytic Reduction Reaction

In order to demonstrate the catalytic hydrogenation of CO_2_, we first tested the Pd@Nb_2_O_5_ samples in a gas‐phase batch photoreactor using ^13^C labeled CO_2_ and H_2_. After 30 min of reaction in the dark under room temperature, negligible amounts of ^13^CO and ^13^CH_4_ products were detected for the Pd@Nb_2_O_5_ samples. This experiment was then carried out using a 300 W Xe lamp focused on the center of the Pd@Nb_2_O_5_ film samples to give a light intensity as high as 4.2 W cm^−2^ and at the same temperature and pressure of the dark reaction. Under illumination, ^13^CO and ^13^CH_4_ were detected as unambiguous products of the CO_2_ reduction reaction, and were confirmed to be carbon‐13 isotopically labeled using gas chromatography–mass spectrometry (Figure S5, Supporting Information). These observations verify that the observed reactions between CO_2_ and H_2_ over the Pd@Nb_2_O_5_ hetero‐nanostructures are light driven. When the batch photoreaction was carried out using pristine Nb_2_O_5_ nanorod samples, only trace amounts of ^13^CO and ^13^CH_4_ were detected under the same illumination and reaction conditions. As shown in Figure 3b, the diffuse reflectance of the Pd@Nb_2_O_5_ samples is much less than that of pristine Nb_2_O_5_ samples throughout the UV, visible, and NIR spectral regions. Additionally, it shows a decreasing trend in diffuse reflectance along with the increase of Pd loading. This phenomenon indicates that a significant amount of light is absorbed by Pd nanocrystals in the Pd@Nb_2_O_5_ samples. Clearly, the presence of Pd nanocrystals enables efficient absorption of light and subsequently causes photoinduced catalytic activity.

**Figure 4:**
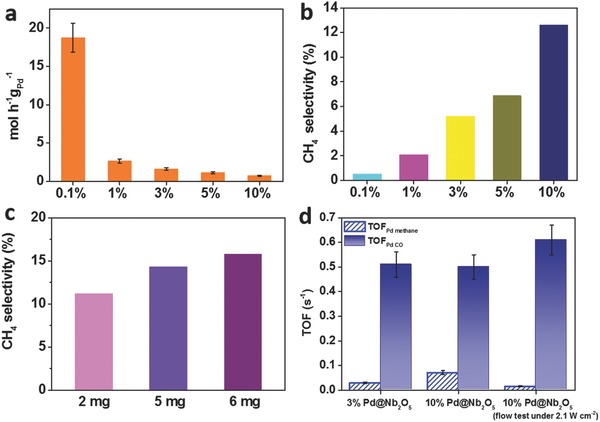
Photothermal catalytic performance of Pd@Nb_2_O_5_ samples. a) CO production rates over Pd@Nb_2_O_5_ film samples with different Pd loadings under irradiation from a 300 W Xe lamp. b) CH_4_ selectivities over Pd@Nb_2_O_5_ film samples with different Pd loadings. c) CH_4_ selectivities over 10% Pd@Nb_2_O_5_ with different sample weights. d) Comparison of TOF_Pd methane_ and TOF_Pd CO_ for 3% Pd@Nb_2_O_5_ and 10% Pd@Nb_2_O_5_ in a batch photoreactor under 4.2 W cm^−2^ and for 10% Pd@Nb_2_O_5_ in flow photoreactor under 2.1 W cm^−2^.

The quantity of products for photocatalytic CO_2_ reduction over different samples was determined using gas chromatography, and the results are plotted in **Figure** **4** and Figures S6–S9 (Supporting Information). Photocatalytic reactions were performed in the light‐limited regime for direct comparison of different samples on reaction efficiency, since the ultimate goal is to optimize performance under conditions where photons are used.^3^ For Pd@Nb_2_O_5_ with 0.1% Pd, we observed a remarkable CO_2_‐to‐CO hydrogenation rate as high as 18.8 mol h^−1^ g_Pd_^−1^ (Figure 4a), and the CH_4_ production rate is also appreciable at 0.1 mol h^−1^ g_Pd_^−1^ (Figure S6, Supporting Information), representing a new benchmark in the champion league of light‐driven gas‐phase CO_2_ reduction. We also achieved a CO production rate of 0.75 mol h^−1^ g_Pd_^−1^ and a CH_4_ production rate of 0.11 mol h^−1^ g_Pd_^−1^ with 10% Pd@Nb_2_O_5_. The selectivity for CH_4_ production exhibits an increasing trend with the increase in Pd loading. The selectivity of the methanation conversion on 10% Pd@Nb_2_O_5_ rises dramatically with an observed 24‐fold increase relative to 0.1% Pd@Nb_2_O_5_ (Figure 4b), pointing to a dependence of the CO_2_ reduction activity and selectivity on Pd nanocrystal size.

To obtain further insights into the activity and selectivity of the photoinduced catalytic process, 10% Pd@Nb_2_O_5_ was evaluated under different intensities of light from the 300 W Xe lamp (Figure S7, Supporting Information). According to the Raman measurements in Figure 3, the local temperature of 5% Pd@Nb_2_O_5_ can be increased dramatically from ambient to as high as 743 K, merely by the photothermal effect of the Pd “nanoheaters.” Hence, it is reasonable to propose that the underlying catalytic mechanism over the Pd@Nb_2_O_5_ hetero‐nanostructures involves the absorption of light and efficient thermalization of absorbed energy, resulting in elevated local temperatures in the Pd@Nb_2_O_5_ catalysts, which lead to the acceleration of the CO_2_ hydrogenation reaction. As shown in Figure S7 (Supporting Information), the reverse water gas shift (RWGS) rates, the methanation rates, and the CH_4_/CO selectivity all increase along with the enhancement of light intensity. This indicates that the selectivity is related to photothermal capability of the samples. Then we investigated the size effect under dark condition with external heating. We conducted dark tests at 160 °C, which is the highest operating temperature we can reach by external heating in our reactor system. We compared the selectivity of two samples (0.5% and 10% Pd), as shown in Figure S8 (Supporting Information). It is concluded that the selectivity toward CH_4_ does increase along with the size of Pd nanocrystals. The selectivity is therefore related to both the local temperature and the size of Pd nanocrystals.

The sample weight dependence of the CH_4_ selectivity was also investigated (Figure 4c), as the selectivity trend mentioned previously could potentially stem from a greater number of active metal sites rather than kinetics. To investigate this possibility, catalyst films containing different weights of the same sample (10% Pd@Nb_2_O_5_) were prepared, and their hydrogenation rates evaluated under identical conditions. If the hydrogenation rates are driven by a photochemical process, in which electron–hole pairs are generated upon light absorption by Nb_2_O_5_, and then transferred to Pd for activating CO_2_, then the CH_4_ selectivity should be independent of the catalyst weight. Nevertheless, the results in Figure 4c indicate that the reaction selectivity is dependent on catalyst weight. Inspection of Figure 4c shows a lower CH_4_ selectivity for a film weighing 2 mg (corresponding to less overall photothermal capability) in contrast to a higher CH_4_ selectivity for a film weighing 6 mg. This weight effect likely originates from an elevated local temperature generated by a larger population of Pd “nanoheaters.” Therefore, the selectivity in hydrogenation is attributable to photothermal‐driven kinetics, and this light‐driven photothermal effect influences the methanation more effectively than the RWGS process.

Another important parameter to consider when designing a catalyst for light‐driven hydrogenation of CO_2_ is its stability, which describes its capacity for long‐term utilization. A repeat of the initial measurement using the 3% Pd@Nb_2_O_5_ catalyst was conducted (Figure S9, Supporting Information), reproducing the rate of around 1.7 mol h^−1^ g_Pd_^−1^. The results demonstrate not only that the 3% Pd@Nb_2_O_5_ catalyst is capable of converting gaseous CO_2_ to CO at an appreciable rate, but also that it is stable under these reaction conditions and can produce rates consistent with the initially measured values even after being irradiated continuously for seven sequential tests.

In general, the activities of photocatalysts reported in the literature degrade with sequential testing for various factors including sintering, photodegradation, catalyst poisoning, and adventitious contamination. By contrast, our photothermal catalysts instead display a slight rate enhancement with the increased testing time. With its superior catalytic performance and long‐term stability, this “nanoheater” photothermal strategy,[[qv: 3a,c]] which can be expanded to include other nanometals with much lower materials cost, is a promising route to scaling up processes for the heterogeneous catalytic hydrogenation of carbon dioxide using renewable solar energy.

## 3 Discussion

In comparison with bulk forms of metal or metal oxide catalysts, nanostructured analogs are expected to provide more active sites to promote catalytic reaction, due to their large surface areas.^12^ To analyze the surface area of Pd@Nb_2_O_5_ samples, Brunauer–Emmett–Teller (BET) measurements were performed, as shown in Table S3 (Supporting Information), indicating that the specific surface areas of these samples are similar in the range 80–90 m^2^ g^−1^. The affinity of a photocatalyst's surface for CO_2_ has also been identified as a critical factor influencing photocatalytic activity.[[qv: 1d,13]] To verify Pd@Nb_2_O_5_ samples' affinity for CO_2_, the CO_2_ capture capacity was determined for each sample using thermogravimetric analysis (TGA). Moreover, the CO_2_ capture capacities of the Pd@Nb_2_O_5_ nanomaterials are also normalized to the surface area of each sample for more accurate comparison. Figure S10 (Supporting Information) reveals that the surface‐area‐normalized CO_2_ capture capacities of pristine Nb_2_O_5_, 0.1%, 0.5%, and 1% Pd, are similar at 0.50, 0.48, 0.47, and 0.48 µmol CO_2_ m^−2^, respectively. There is a notable decrease in the normalized CO_2_ capture capacity for samples with Pd loading beyond 3%, which could be ascribed to the reduced amount of exposed Nb_2_O_5_ surface, suggesting that Pd does not assist with the affinity of CO_2_ for reaction with hydrogen.

In this work, we have been able to demonstrate a highly efficient and stable photothermal‐catalytic system with tunable selectivity for the reduction of CO_2_. To compare the catalytic activity more accurately, we calculated the TOF for CO and CH_4_ production as a function of active surface Pd sites, which was determined via selective CO chemisorption; these results are shown in Figure 4d and Table S4 (Supporting Information). When the reaction was catalyzed in a batch reactor with an illumination intensity of 4.2 W cm^−2^, the TOF_Pd CO_ values for the 3% and 10% Pd samples were similar at 0.51 and 0.50 s^−1^, respectively, while the TOF_Pd methane_ value for the 10% Pd sample (0.073 s^−1^) is 2.4 times higher than that of the 3% Pd sample (0.03 s^−1^).

The photocatalytic CO_2_ hydrogenation properties of the Pd@Nb_2_O_5_ samples were also evaluated in a light‐powered flow photoreactor, which is relevant to more practical operating conditions in industry. In the light‐flow system, we achieved a high gas‐phase light CO production rate of 0.91 mol h^−1^ g_Pd_^−1^ and TOF_Pd CO_ as high as 0.61 s^−1^ over a 10% Pd@Nb_2_O_5_ sample with illumination intensities of 2.1 W cm^−2^.

We also evaluated the energy conversion efficiency of these photoreactions in both batch‐ and flow‐type reactors for gas‐phase CO_2_ reduction. As shown in Table S5 (Supporting Information), 0.23% energy conversion efficiency is achieved in the flow system over 10% Pd@Nb_2_O_5_, while 0.05% energy conversion efficiency is obtained in the batch system over 0.1% Pd@Nb_2_O_5._ Notably, even at these low loadings of Pd, photothermal hydrogenation of CO_2_ still shows a leading‐edge efficiency when compared with literature examples. With materials and process engineering optimization, the performance metrics of these hetero‐nanostructured catalysts can likely be further enhanced and, with the use of more earth‐abundant elements, the cost of the process can be reduced.

**Figure 5:**
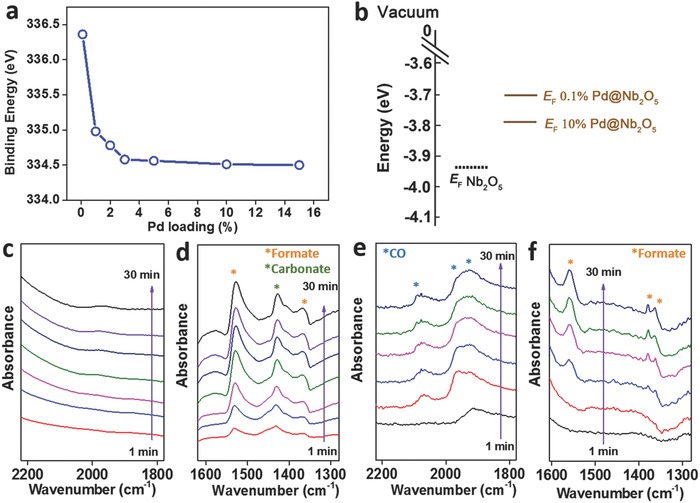
XPS, UPS, and in situ DRIFT analysis: a) Pd 3d_5/2_ binding energy position as a function of Pd loading. b) The Fermi‐level position of pristine Nb_2_O_5_ in comparison with that of 0.1% Pd@Nb_2_O_5_ and 10% Pd@Nb_2_O_5_ measured by UPS (Fermi levels are referenced to the vacuum level, i.e., the materials' work functions). c,d) In situ DRIFT spectra of 0.1% Pd@Nb_2_O_5_ under a gas mixture of H_2_ and CO_2_ (1:1 ratio) at 240 °C. e,f) In situ DRIFT spectra of 10% Pd@Nb_2_O_5_ under a gas mixture of H_2_ and CO_2_ (1:1 ratio) at 240 °C.

To investigate the elemental composition, bonding information, and electronic properties of the samples, X‐ray photoelectron spectroscopy (XPS) and ultra‐violet photoemission spectroscopy (UPS) measurements were performed. Figure S12 (Supporting Information) illustrates the Pd 3d core level XPS spectra of the set of Pd@Nb_2_O_5_ samples, from which we clearly observed the 3d_5/2_ and 3d_3/2_ peaks shifted from 334.5 and 340 eV, respectively, for 15% Pd to 336.4 and 342 eV, respectively, for 0.1% Pd. Such a shifting is plotted as a function of Pd loading and shown in **Figure** **5**a.

An electron donor–electron acceptor interaction model can be used to explain the shift in Pd binding energy; at the interface between the Pd and the Nb_2_O_5_, electrons are transferred from the initially d electron‐rich metal (Pd) to the metal oxide (Nb_2_O_5_), which has empty states in its conduction band. Charge transfer of this type can lead to a positively charged Pd surface.^5^ To substantiate this proposal, UPS measurements have been conducted on the pure Nb_2_O_5_ sample, 0.1% Pd sample, and 10% Pd sample to measure their respective work functions, which dictate the position of the Fermi level with reference to the vacuum level. As shown in Figure 5b, the Fermi levels *E*_f_ of composite Pd@Nb_2_O_5_ are located less negatively compared with that of pristine Nb_2_O_5_. This implies that at the heterojunction between Pd and Nb_2_O_5_, electrons will flow from Pd to Nb_2_O_5_ to equilibriate the chemical potential, resulting in the formation of positively charged Pd atoms.

With this model, the positively charged Pd surface can affect the strength of binding of adsorbed reactants, intermediates, and products.^14^ This change of the adsorption strength to the Pd nanocrystal surface is a key factor in determining the reactivity and selectivity of the CO_2_ hydrogenation reaction. Notably, *E*_f_ of the 0.1% Pd sample locates closer to the vacuum level compared with that of the 10% Pd sample; thus, more charge transfer from Pd to Nb_2_O_5_ would take place with the former compared to the latter.

To obtain further insights into the surface chemistry responsible for CO_2_ reduction on this Pd@Nb_2_O_5_ catalyst, a series of in situ diffuse reflectance infrared Fourier transform spectroscopy (DRIFTS) measurements was performed in a flow cell under reaction operando conditions.^15^ In the spectra recorded over 0.1% Pd@ Nb_2_O_5_ (Figure 5c,d), the bands at 1530 and 1363 cm^−1^ were assigned to a formate intermediate species, with the band at 1427 cm^−1^ being indicative of a monodentate surface carbonate.[[qv: 15a,16]] For 10% Pd@ Nb_2_O_5_ (Figure 5e,f)_,_ the bands at 1562, 1375, and 1361 cm^−1^ represent diagnostic vibrational modes of an adsorbed formate intermediate, while the bands at 2085 and 1965 cm^−1^ appear to signal vibrational fingerprints of surface absorbed CO.[[qv: 15a,16]] Hence, formate and monodentate surface carbonate species, which are intermediates in CO_2_‐to‐CO hydrogenation, are identified over the low Pd loading samples. In this case, no obvious evidence of CO intermediate species is observed, indicating easy desorption of CO. By contrast, samples with larger Pd loading not only hydrogenate CO_2_ to formate and then to CO, but can efficiently retain CO intermediate species on their surfaces, where it can be further hydrogenated into CH_4_. By identifying the intermediate species present on the surface of the Pd@Nb_2_O_5_ catalysts, these DRIFTS results reveal the mechanistic details that control the corresponding selectivity for production of CO versus CH_4_.

**Figure 6:**
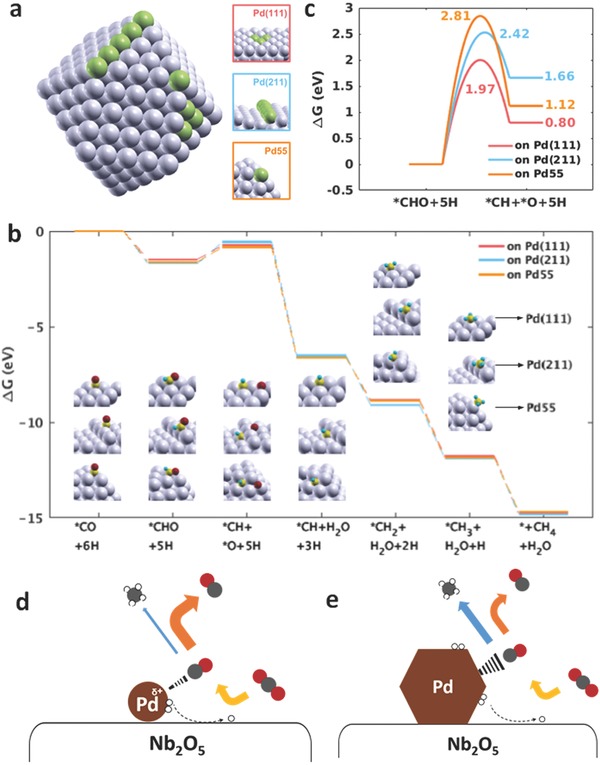
Density functional theory analysis and reaction mechanism on low Pd loading and high Pd loading samples. a) Pd(111), stepped Pd(211), and Pd55 computational models for representing surface terrace sites, edge and corner sites. b) Calculated free energy diagram for *CO intermediate reduction to CH_4_ on Pd(111), Pd(211), and Pd55. c) Calculated activation barrier for the rate‐determine step on Pd(111), Pd(211), and Pd55. d) Schematic illustration depicts the weak CO bonding of small Pd nanocrystals in low Pd loading sample, leading to mainly CO production. e) Schematic illustration depicts the strong CO bonding of large Pd nanocrystals in the high Pd loading sample, leading to CH_4_ production.

Density functional theory (DFT) calculations were conducted to rationalize the size effect of Pd nanocrystals on the CO_2_ to CO versus CH_4_ selectivity. Previous studies have shown the conversion of gas‐phase CO_2_ to CO to occur primarily at the metal–metal oxide interface, and the subsequent hydrogenation of CO to CH_4_ to occur at the metal sites.[[qv: 15a]] However, while the hydrogenation steps involved in the conversion CO_2_ to CO have been extensively studied,^17^ a more detailed understanding of the CO hydrogenation mechanism at metal nanocrystal sites is necessary in order to address the issue of CO versus CH_4_ selectivity. DFT calculations were therefore conducted to investigate the production of CH_4_ via the *CO intermediate. The Pd(111), Pd(211) surfaces, and a model Pd55 nanocrystal were selected to model terrace, edge, and corner sites, respectively (**Figure** **6**a). The most stable intermediate configuration was computed for each step in the hydrogenation of *CO to CH_4_ by testing all possible adsorption sites on the corresponding surface at each step (Tables S6 and S7, Supporting Information). Figure 6b shows nearly all hydrogenation steps, including those where hydrogen atoms are added to the intermediates, to have an exothermic energy of reaction on both the Pd(111), Pd(211) surfaces, and the Pd55 cluster, with the exception of the endothermic step associated with the bond cleavage of C—O in the *CHO intermediate. This suggests the *CHO → *CH + *O step to be the rate‐determining step in the reaction pathway, which is supported by similar findings in the theoretical literature.^18^ A comparison of the CO and CH_4_ production pathways was recently executed in details, which agrees with mechanistic assertions in this study.^18^ Nudged elastic band (NEB) calculations were conducted to determine the associated activation barriers and transition states. The results (Figure 6c) revealed the Pd(111) surface to have the lowest activation barrier (1.97 eV), followed by Pd(211) (2.42 eV) and Pd55 (2.81 eV), which suggests the terrace site, analogous to the Pd(111) surface, to be the most favorable site for methanation of CO_2_ on Pd nanocrystals.

Based on the combination of all the experimental and theoretical results presented herein, the favored surface reaction pathway for the photothermal catalytic hydrogenation of CO_2_ to CO versus CH_4_ on Pd@Nb_2_O_5_ is presented in Figure 6c,d. The significant photothermal effect of Pd nanocrystals, driven by efficient light absorption and light‐to‐heat conversion, leads to an elevated local temperature of the Nb_2_O_5_ support, which drives the CO_2_ hydrogenation reaction via the formate pathway. As a result of electron donation from Pd to the Nb_2_O_5_ support, the Pd nanocrystals develop a positive surface charge that is more pronounced for the smaller Pd nanocrystals (Figure 6c). The size of the Pd nanocrystals also determines the percentage of terrace/edge/corner sites. To quantify the relation between nanoparticle sizes and surface site geometries, Pd nanocrystals of different sizes were modeled by octahedral clusters of atoms, and their respective terrace/edge/corner site distribution was plotted as a function of size (Figure S15, Supporting Information). Indeed, as the nanocrystal size becomes larger, the number of edge and corner sites decreases, while the number of terrace sites increases; therefore, terrace sites, analogous to the Pd(111) facets, are more dominant on the surface of larger nanoparticles.^6^ This fact illustrates why larger Pd loadings, characterized by an increased presence of surface terrace sites, which exhibit a lower activation barrier to C—O cleavage, are most favorable toward methanation. When the loadings and sizes of Pd nanocrystals are small (Figure 6c), and the surface local positive charge facilitates the desorption of CO,[[qv: 14d–f]] the activation barrier for the methanation is higher, thereupon favoring highly efficient and selective production of CO. By contrast, for large loadings and sizes of Pd nanocrystals (Figure 6d), a lower surface positive charge, more methanation favorable terrace sites, and a more efficient photothermal effect, combine to assist in the adsorption and hydrogenation of the intermediate species *CO to favor CH_4_.

The reaction scheme for the overall mechanism is shown in Figure S16 (Supporting Information). The conversion of CO_2_ to CH_4_ can be divided into three steps. In the first step, CO_2_ is hydrogenated to CO at the interface of Pd and Nb_2_O_5_. In the second step, the intermediate CO species are adsorbed onto Pd nanocrystals. In the third step, the adsorbed CO intermediate species are converted into CH_4_ at the surface, edge, or corner sites of Pd nanocrystals. The more positive surface charge on the smaller Pd nanocrystals will influence the CO adsorption step. A dynamic restructuring phenomenon of the niobium oxide support could also occur under CO_2_ operating conditions that modify reactivity.^6^ This effect is of general importance and pertinent to many forms of metal‐oxide‐based heterogeneous catalysis.

Alternative reaction mechanisms may also be responsible for the observations described above.^19^ For example, H spilled over from the dissociation of H_2_ on Pd can form coordinated formate (HCO_2_) on the surface of Nb_2_O_5_, located at the interface between Pd and Nb_2_O_5_. A surface acetal (H_2_CO_2_) could then be formed via transfer of a second H from Pd to the coordinated formate. Subsequent reaction of the acetal could involve a concerted transfer of H from the Pd to the acetal to form a surface methoxide (CH_3_O) and surface hydroxyl (OH) groups. The methoxide could react further with H spilled over from the Pd to form CH_4_ and H_2_O that then desorb from the surface of the Nb_2_O_5_ nanorods. It is also possible to consider the Nb_2_O_5_ surface sites that capture CO_2_ molecules to be active sites in the CO_2_ conversion. We calculated the TOF for CO and CH_4_ production as a function of active surface Nb_2_O_5_ active sites, which was determined by multiplying the CO_2_ capture capacity by the surface area of each sample; these results are shown in Table S4 (Supporting Information).

In closing, it is worth commenting on a likely cause of the extraordinary long‐term stability observed for the Pd@Nb_2_O_5_ catalyst in the photothermal hydrogenation of CO_2_, mentioned earlier and displayed graphically (Figure S17, Supporting Information). Recall that thermal reactions on nanostructured catalysts often suffer from deactivation caused by loss of surface area induced by sintering. This is not found to be the case for the photothermal hydrogenation of CO_2_ where the size of the Pd nanocrystals remains essentially unchanged before and after the catalyst testing. It seems that in the case of the photothermally driven heterogeneous catalytic hydrogenation of CO_2_ described in this study, where the active palladium “nanoheaters” can achieve local temperatures that are high enough to drive the conventional thermal reactions, the heating and surface chemistry is spatially confined to the interface between the Pd nanocrystals and the Nb_2_O_5_ nanorods, thereby unable to cause the macroscopic heating of the entire nanostructured catalyst required to cause sintering, loss of surface area, and lack of stability. To delve deeper into this proposal would require first principles quantum and classical mechanical analyses of the effect of size on heat diffusion and transfer from the nanoscale to the macroscale,^20^ to see if heat transport processes of the type envisioned are blocked between the Pd nanocrystal and the Nb_2_O_5_ nanorod.

## 4 Conclusion

Photothermal engineering of the catalytic hydrogenation of CO_2_ on Pd@Nb_2_O_5_ has been shown to be an effective strategy for optimizing the activities and selectivities, stabilities, and efficiencies of the reverse water gas shift and methanation reactions, taking their performance metrics into the champion league of catalysts. It is envisioned that this approach will work equally well for earth‐abundant and low‐cost M@MO*_x_* hetero‐nanostructures, an advance that speaks well for their implementation in future solar‐powered CO_2_ refineries.

## 5 Experimental Section

*Preparation of Nb_2_O_5_*: A mass of 340 mg niobium powder (25 Mesh, Aldrich) was dissolved in a hydrochloric acid solution (4.5 mL HCl, Sigma–Aldrich; 5 mL deionized water) in a Pyrex beaker, which was then stirred and ultrasonicated for half an hour. The solution was subsequently transferred into a 50 mL Teflon‐lined stainless steel autoclave, and the hydrothermal reaction was performed at *T* = 200 °C for 24 h. After the hydrothermal synthesis, the obtained product was washed three times with deionized water, and the powder was dried in a vacuum oven at *T* = 70 °C for 12 h.

*Preparation of Pd@Nb_2_O_5_ Hetero‐Nanostructure*: Nanostructured Pd@Nb_2_O_5_ samples with different loadings of Pd were synthesized via a microwave‐assisted reaction. In a typical microwave synthesis, 50 mg of nanorods was suspended in anhydrous ethanol (20 mL), in a Pyrex vessel with 40 mL capacity. A stock solution of Na_2_PdCl_4_ (Na_2_PdCl_4_·3H_2_O, Alfa Aesar) in anhydrous ethanol (20 mL) was then prepared with a concentration of 1 mg mL^−1^. A certain amount of Pd precursor solution was added to the dispersion, which was then sonicated for 30 min. Subsequently, the vessel was capped and transferred to the microwave reactor (CEM Discover, 220 W, 220 psi, *T* = 150 °C, 20 min). After being washed with deionized water for three times, the sample was dried into a vacuum oven at *T* = 70 °C for 12 h, followed by a post‐treatment at 120 °C in air for 24 h to obtain the Pd@Nb_2_O_5_ nanostructured samples.

*Characterization*: The morphologies of the Pd@Nb_2_O_5_ catalysts were assessed using a Hitachi H‐7000 transmission electron microscope at 100 kV. PXRD patterns were obtained using a Bruker D2‐Phaser X‐ray diffractometer with Cu Kα radiation at 30 kV. The amount of Pd in the hetero‐nanostructures was detected using ICP−AES (Thermo Electron Corp. Adv. ER/S). Prior to the ICP measurements, the hetero‐nanostructures were immersed in concentrated nitric acid with agitation for 12 h to dissolve the Pd@Nb_2_O_5._To determine the surface area, Pd@Nb_2_O_5_ catalysts were outgassed overnight at 80 °C and then analyzed by volumetric nitrogen adsorption at 77 K using a Quantachrome Autosorb‐1‐C. The surface area was calculated based on the BET theory. XPS was performed using a Perkin Elmer Phi 5500 ESCA spectrometer in an ultrahigh vacuum chamber with a base pressure of 1 × 10^−9^ Torr. The spectrometer used an Al Kα X‐ray source operating at 15 kV and 27 A. Calculations were made by fitting C 1s peak to 284.5 eV, and samples were prepared by compacting a uniform layer of the materials on a carbon tape. During the measurement, survey and high‐resolution C 1s, O 1s, Nb 3d, and Pd 3d spectra were recorded. All data analyses were carried out using the Multipak fitting program.

HRTEM images were acquired using the aberration‐corrected JEOL JEM‐ARM200CF transmission electron microscope operated at 200 keV. With a spherical aberration corrector, the TEM images can achieve 0.11 nm spatial resolution.

CO_2_ adsorption capacity was determined using thermogravimetric analysis (Discovery TGA, TA Instruments). Each sample (*≈*5 mg) was first heated at a rate of 10 °C min^−1^ to 120 °C under a nitrogen flow of 100 mL min^−1^. This condition was maintained for 1 h to remove any adsorbed moisture. Subsequently, the gas flow was switched to carbon dioxide at 100 mL min^−1^ under the same temperature for 2 h. The weight gain between the N_2_ and CO_2_ gas streams was directly used for CO_2_ capacity calculation, which was normalized to the surface area of each sample.

Ultraviolet photoemission spectroscopy was performed using a PHI 5500 multitechnique system. The samples were prepared by drop‐casting the suspension of the materials onto silicon wafers. The measurement was conducted under a pressure of ≈10^−8^ Torr. UV radiation was produced from He Iα (21.22 eV), and a bias of −15 V was applied to measure the spectrum.

X‐ray absorption spectroscopy (XAS) analysis of the Pd nanoparticles and reference materials was performed using the Sector 20‐BM beamline of the advanced Photon Source at Argonne National Laboratory in Argonne, IL, USA. XAS spectra were obtained from reference foils and powders in transmission detection mode, while the spectra for Nb_2_O_5_‐supported Pd nanoparticles were acquired using transmission detection for the Nb K‐edge and fluorescence detection for the Pd K‐edge. Samples were cooled to 90 K using a cryostatic sample holder in order to suppress thermal lattice vibrations, thereby enhancing the intensity of the observed EXAFS signal intensity. X‐ray absorption near edge structure (XANES) spectra were background‐subtracted and normalized for more accurate comparison, with Nb and Pd metal foils being used to energy‐calibrate each spectrum. Data processing and EXAFS fitting were performed using WinXAS software^21^ in conjunction with scattering path functions generated using FEFF^22^ and crystal structures obtained from the Crystallographic Open Database.^23^ With the exception of the PdO reference material, Pd EXAFS fits were performed using a selected region of interest corresponding to Pd–Pd single scattering paths of *≈*2.75 Å in length. For PdO, direct Pd–O and Pd–Pd single scattering paths of *≈*2.02 and 3.05 Å in length were included in the region of interest. The slight difference between the Pd 0.1% sample and other supported Pd nanoparticles was attributed to noise in the XAS data, due to the low relative concentration of Pd in this sample. It should also be considered that the presence of surface defects can decrease the observed CNs obtained from EXAFS fitting, and so this might also contribute to these low numbers. Likewise, the gradually increasing Debye–Waller factor (σ^2^) values could be equally attributable to the small size or increased structural disorder of the Pd nanoparticles. The slightly shorter Pd—Pd bond length of the 0.1% Pd sample was, however, indicative of very small nanoparticles, especially in conjunction with the observed CN values.

Diffuse reflectance spectra of the samples were determined using a Lambda 1050 UV/Vis/NIR spectrometer from Perkin Elmer, which was equipped with an integrating sphere with a diameter of 150 mm. The samples for analysis were films prepared by drop‐casting aqueous dispersions of Pd@Nb_2_O_5_ and Nb_2_O_5_ samples onto borosilicate glass microfiber filters, and the weight of powder on each film was ≈2 mg.

CO chemisorption was measured using an Autosorb iQ analyzer (Quantachrome Instruments). As for pretreatment, each sample (200 mg to 1 g) was first heated at a rate of 5 °C min^−1^ to 120 °C under vacuum, and this condition was maintained at 120 °C for 5 h. Subsequently, the gas flow was switched to H_2_ and the sample was heated at 10 °C min^−1^ until the temperature reached 250 °C. This condition was maintained for 2 h under H_2_ flow prior to be evacuated for 2 h. Then the sample was cooled down to 40 °C. The vacuum–volumetric isotherm measurement was then performed at 40 °C. The combined amount of physisorbed and chemisorbed CO was first measured, then, a vacuum was pulled on the sample to remove the physisorbed CO while the chemically adsorbed CO remained bound to the metal. A second isotherm was measured which represented only the physisorbed amount of CO. The physisorbed isotherm was then subtracted from the combined isotherm to determine the chemisorbed isotherm.

In situ DRIFT spectra were collected on a Thermo Scientific iS50 series Fourier transform infrared (FT‐IR) instrument equipped with a Harrick Praying Mantis DRIFT accessory. Each spectrum was recorded at 4 cm^−1^ resolution, using an average of 64 scans. Powder samples were placed into the holder without packing or dilution. A background IR spectrum was taken following 1.5 h of purging under He at 150 °C. Different mixtures of gases were then introduced into the cell, with flow rates controlled by electronic mass flow controllers. In all transient experiments, 5% of H_2_ diluted in He was first introduced for 20 min, followed by 15 min of pure He. Pure CO_2_ was then introduced into the cell, again followed by 15 min of He. Finally, a mixture of H_2_ and CO_2_ (2.5% H_2_, 50% CO_2_, 47.5% He) was flowed for 30 min. A total flow rate of 20 mL min^−1^ was used for all gas mixtures, and a temperature of 240 °C was maintained for all experiments. DRIFT spectra were recorded every ≈90 s when a new gas mixture was introduced, then every ≈5–10 min after 10 min time durations.

*Gas‐Phase Catalytic Measurements—Batch System*: The batch‐system‐based gas‐phase catalytic measurements were carried out in a custom‐built 12 mL stainless steel batch reactor with a fused silica view port sealed with a Viton O‐ring.

In a typical gas‐phase test, 2 mg of samples was deposited onto a borosilicate filter support having an area of 1.2 cm^2^ (the illumination area was 1 cm^2^), which was then loaded into a custom‐built stainless steel batch reactor with a volume of 12 mL, followed by filling the reactor with CO_2_ and H_2_ gas at 1:1 ratio to a total pressure of 27 psi. In order to confirm with complete certainty that the products stem from reactant CO_2_ gaseous molecule rather than adventitious carbon sources, isotope‐tracing experiments were carried out using ^13^CO_2_ (99.9 atomic %; Sigma–Aldrich). The filter with the catalyst was then loaded into the reactor, which was evacuated using an Alcatel dry pump prior to being purged with H_2_ (99.9995%) at a flow rate of 20 mL min^−1^. The reactor was first evacuated and then injected with ^13^CO_2_ followed by H_2_ at a 1:1 ratio to a total pressure of 27 psi prior to being sealed. An Omega PX309 pressure transducer was used to monitor the presser inside the reactor. Samples were irradiated using a 300 W Xe lamp for durations of 30 min. No external heating source was connected to the reactor during the photothermal gas‐phase test. The analysis of product gases was conducted with a flame ionization detector (FID) installed in an SRI‐8610 gas chromatograph (GC) with a 6′ Haysep D column. Isotopically labeled product gases were measured using an Agilent 7890A gas chromatographic mass spectrometer with a 60 m GS‐CarbonPLOT column fed to the mass spectrometer.

*Gas‐Phase Catalytic Measurements—Flow System*: The photothermal reduction of CO_2_ by H_2_ was carried out in a cylindrical flow reactor modeled as a continuously stirred tank reactor of volume ≈12 mL. A 300 W Xe lamp (Newport) was used as the light source for the reaction. The measured intensity was *≈*2.1 W cm^−2^. CO_2_ and H_2_ flow rates were controlled between 0.1 and 2 sccm by two mass flow controllers (Alicat MC‐2SCCM‐D/5M). After leak testing and evacuation of the reaction system, CO_2_ (Grade 5.0) and H_2_ (Grade 5.5) of ratio 1:1–1:4 were introduced. The concentrations of CO and CH_4_ products in the effluent gas were periodically sampled and analyzed by a gas chromatograph (SRI 8610) with an FID using He as the carrier gas.

*Gas‐Phase Catalytic Measurements—Theoretical Methods*: DFT calculations were conducted using the plane‐wave basis in the Quantum Espresso software.^24^ All calculations were carried out using ultrasoft pseudopotential and Perdew–Burke–Ernzerhof exchange–correlation functions.^25^ Kinetic energy cutoffs of 50.0 and 350.0 Ry were used for wave functions and charge densities, respectively. For Pd(111), Pd(211), and their interactions with substrates, k‐points were sampled using a 4 × 4 × 1 grid during the Brillouin zone integration. Because of the large size of the Pd55 nanoparticle model, Brillouin zone integrations were performed at the gamma k‐point only. All Brillouin zone integrations were performed using Gaussian smearing over a width of 0.02 Ry. During geometric relaxations, a convergence threshold of 10^−6^ Ry was used, and Davidson iterative diagonalization algorithm was employed. All calculations were spin‐polarized with the local‐spin‐density approximation.

The bulk structure of palladium was first relaxed using a supercell containing 32 Pd atoms, giving a lattice constant of 3.9425 Å, which was in good agreement with other literature values.^26^ This relaxed lattice constant was then employed to construct the Pd(111), Pd(211), and Pd55 models (Figure S13, Supporting Information). For Pd(111) modeling, the supercell consisted of four 3‐by‐3 layers of Pd atoms, while for the Pd(211) modeling, four 3‐by‐4 layers of Pd atoms were used for the surface unit cell. A vacuum distance of 20.5 Å was used for both models. During geometrical optimizations on the Pd(111) and Pd(211) surfaces, the bottom two layers were fixed, while the upper two layers and the substrates were allowed to relax. For Pd55 nanoparticles, a unit cell of 17 Å × 17 Å × 17 Å was used to separate neighboring nanoparticles.

Transition state information was obtained using NEB calculations with the climbing image method.^27^ The initial and final images were first relaxed using the parameters previously described. The NEB calculations were carried out using 11 images for each step of iteration. To reduce the computational burden incurred, k‐point samplings were reduced to 2 × 2 × 1 for Pd(111) and gamma point for Pd(211) and Pd55 during their Brillouin zone integrations.

To verify the DFT results, the adsorption energies and transition state energies were compared with other literature values. The enthalpy of adsorption (*E*_ads_) was defined by

(1) $$E_{\mathrm{ads}}\;\;=\;\;E_{\mathrm{adsorbate/surface}}-(E_{\mathrm{adsorbate}}+E_{\mathrm{surface}})$$

where *E*_adsorbate/surface_ is the total energy of the surface containing the adsorbed species, *E*_adsorbate_ is the total energy of the free adsorbate (in the gas phase at noninteracting distance), and *E*_surface_ is the total energy of either the bare surfaces or cluster. By this definition, negative adsorption enthalpies indicate exothermic adsorption. The adsorption energies of *H (−2.853 eV), *CO (−1.995 eV), and *OH (−2.461 eV) on Pd(111) closely matched those from other study (−2.909, −2.051, and −2.622 eV).^28^ The transition state energy of C—O bond cleavage on Pd(111) (1.97 eV) and its reaction energy (0.80 eV) closely matched those from study (1.96 and 0.81 eV).^18^

To obtain the active site distribution in Figure S15 (Supporting Information), a MATLAB script was written to construct octahedral NPs of different diameters. The script was written in such a way that the final NP had the same face‐centered cubic internal structure as bulk Pd and was externally enclosed by (111) and (100) planes to give an external octahedral structure. The atoms that belong to bulk, terrace, edge, and corner sites were then counted. These sites were defined by their coordination numbers, with bulk atoms having the largest coordination number (12) and corner atoms having the smallest (5).

## Conflict of Interest

The authors declare no conflict of interest.

## Supporting information

Supplementary
