# Supplementary material for: Photothermal Catalyst Engineering: Hydrogenation of Gaseous CO2 with High Activity and Tailored Selectivity
Source: Adv Sci (Weinh). 2017 Jul 25;4(10):1700252. doi: 10.1002/advs.201700252 (PMC5644230; doi:10.1002/advs.201700252)
Supplement: Supplementary file 1 — Supplementary [file ADVS-4-na-s001.pdf]

Copyright WILEY-VCH Verlag GmbH & Co. KGaA, 69469 Weinheim, Germany, 2017.

ADVANCED SCIENCE Open Access

Supporting Information

for *Adv. Sci.,* DOI: 10.1002/advs.201700252

Photothermal Catalyst Engineering: Hydrogenation of Gaseous CO_2 with High Activity and Tailored Selectivity

*Jia Jia, Hong Wang, Zhuole Lu, Paul G. O'Brien, Mireille Ghoussoub, Paul Duchesne, Ziqi Zheng, Peicheng Li, Qiao Qiao, Lu Wang, Alan Gu, Abdinoor A. Jelle, Yuchan Dong, Qiang Wang, Kulbir Kaur Ghuman, Thomas Wood, Chenxi Qian, Yue Shao, Chenyue Qiu, Miaomiao Ye, Yimei Zhu, Zheng-Hong Lu, Peng Zhang, Amr S. Helmy, Chandra Veer Singh, Nazir P. Kherani, Doug D. Perovic, and Geoffrey A. Ozin**

Copyright WILEY-VCH Verlag GmbH & Co. KGaA, 69469 Weinheim, Germany, 2017.

Supporting Information

Article type: Full Paper

Title : Photothermal Catalyst Engineering: Hydrogenation of Gaseous CO_2 with High Activity and Tailored Selectivity

*Jia Jia, Hong Wang, Zhuole Lu, Paul G. O'Brien, Mireille Ghoussoub, Paul Duchesne, Ziqi Zheng, Peicheng Li, Qiao Qiao, Lu Wang, Alan Gu, Abdinoor A. Jelle, Yuchan Dong, Qiang Wang, Kulbir Kaur Ghuman, Thomas Wood, Chenxi Qian, Yue Shao, Chenyue Qiu, Miaomiao Ye, Yimei Zhu, Zheng-Hong Lu, Peng Zhang, Amr S. Helmy, Chandra Veer Singh, Nazir P. Kherani, Doug D. Perovic, and Geoffrey A. Ozin**

J. Jia, P. Li, K. K. Ghuman, C. Qiu, Prof. Z. H. Lu, Prof. C. V. Singh, Prof. N. P. Kherani, Prof. D. D. Perovic

Department of Materials Science & Engineering, University of Toronto, 184 College Street, Toronto, Ontario, M5S 3E4, Canada

H. Wang, M. Ghoussoub, Z. Zheng, L.Wang, A.Gu, A. A. Jelle, Y. Dong, C. Qian, Y.Shao, Prof. G. A. Ozin

Materials Chemistry and Nanochemistry Research Group, Solar Fuels Cluster, Department of Chemistry, University of Toronto, 80 St. George Street, Toronto, Ontario, M5S 3H6, Canada,

Email: gozin@chem.utoronto.ca

Z. Lu, T.Wood,

Department of Chemical Engineering & Applied Chemistry, University of Toronto, 200 College Street, Toronto, Ontario, M5S 3E5 Canada

Prof. P. G. O’Brien,

Department of Mechanical Engineering, Lassonde School of Engineering, York University, Toronto M3J 1P3, Canada

P. Duchesne, Prof. P. Zhang,

Department of Chemistry, Dalhousie University, 6274 Coburg Road, P.O. Box 15000, Halifax, Nova Scotia B3H 4R2, Canada

Q. Qiao, Prof. Y. Zhu,

Condensed Matter Physics and Materials Science Department, Brookhaven National Laboratory, Upton, New York 11973, United States

Q.Qiao,

Department of Physics, Temple University, Philadelphia, Pennsylvania 19122, United States

Q. Wang,

State Key Laboratory of Coal Conversion, Institute of Coal Chemistry, The Chinese Academy of Sciences, Taiyuan 030001 People's Republic of China

Prof. M. Ye,

Zhejiang Key Laboratory of Drinking Water Safety and Distribution Technology, Zhejiang University, Hangzhou, 310058, China

Prof. A. Helmy, Prof. N. P. Kherani,

Department of Electrical and Computing Engineering, University of Toronto, 10 King's College Road, Toronto, Ontario, M5S 3G4, Canada

Corresponding author: Geoffrey A Ozin

E-mail: gozin@chem.utoronto.ca

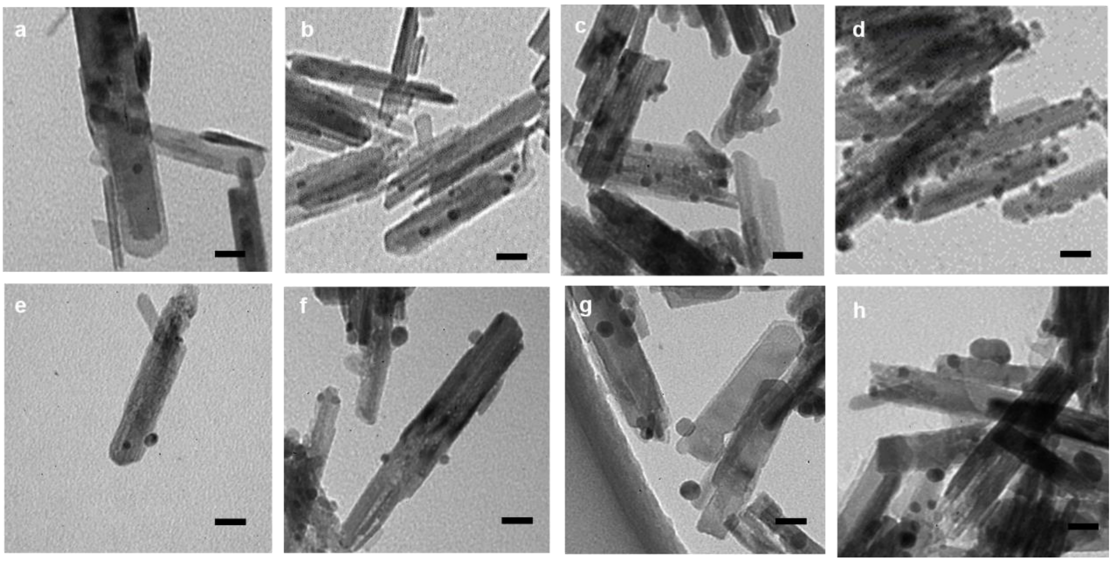

Figure S1. TEM bright field images of a) 0.1%Pd@Nb_2O_5, b) 0.5% Pd@Nb_2O_5, c) 1% Pd@Nb_2O_5, d) 2% Pd@Nb_2O_5, e) 3% Pd@Nb_2O_5, f) 5% Pd@Nb_2O_5, g) 10% Pd@Nb_2O_5 and h) 15% Pd@Nb_2O_5. Scale bar: 20 nm.

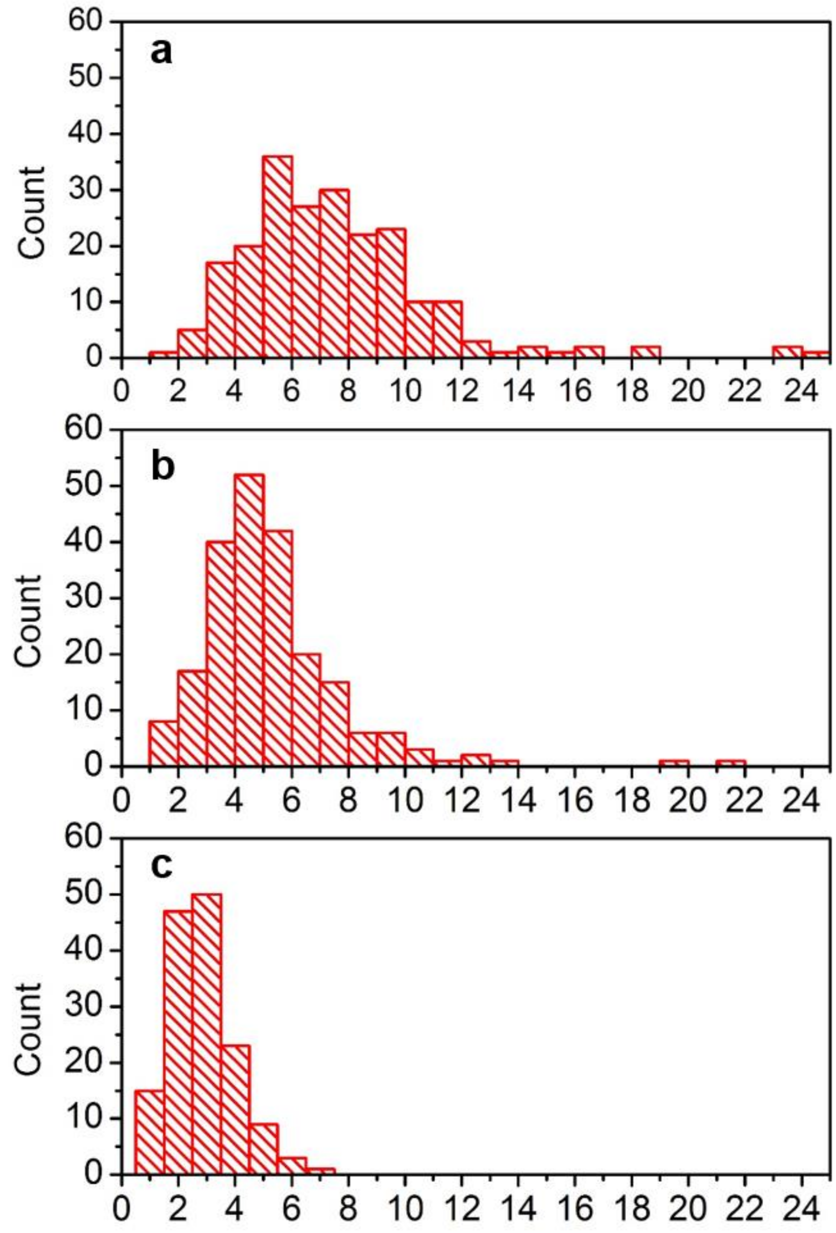

Figure S2. Pd particle size distribution determined for a) 10% Pd@Nb_2O_5, b) 3% Pd@Nb_2O_5, and c) 0.1% Pd@Nb_2O_5 from TEM images.

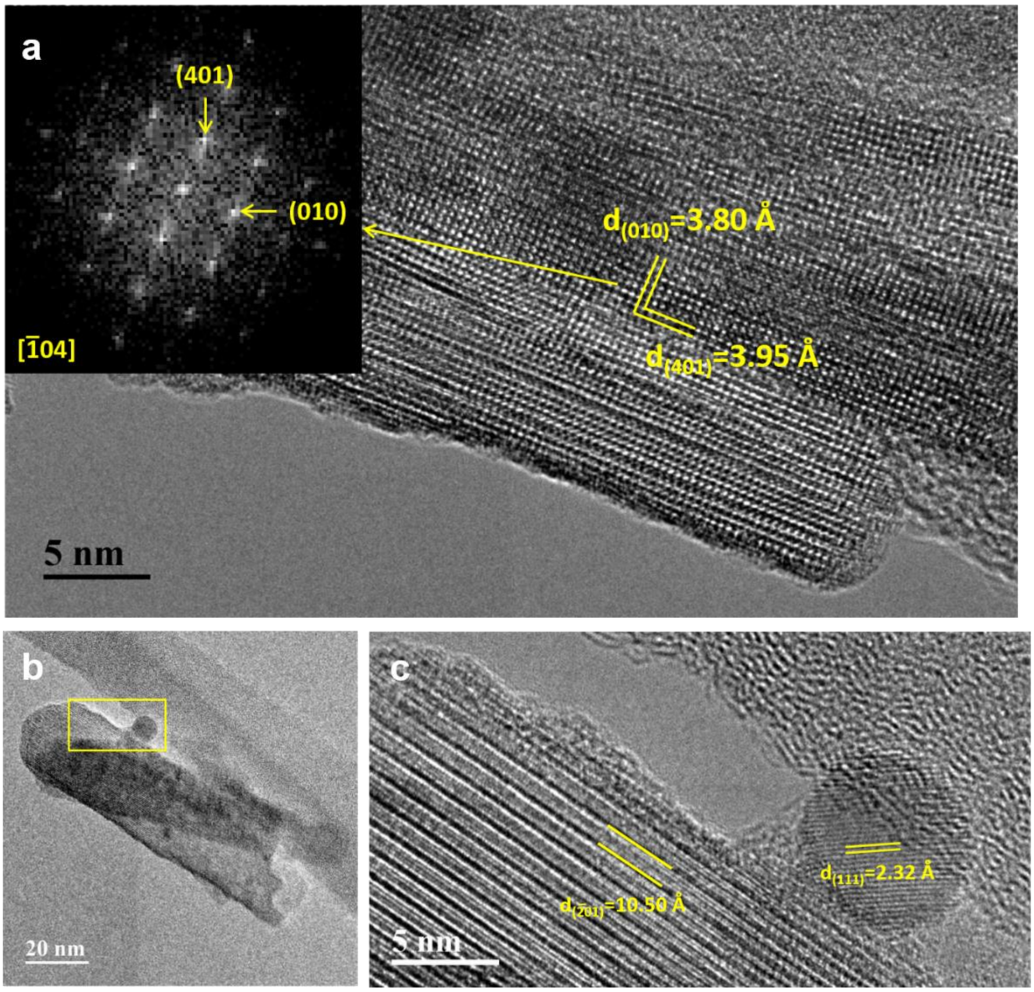

Figure S3. a) HRTEM images of Nb_2O_5 nanorod viewed from its [$\bar{1}$ 0 4] direction, showing (010) and (401) planes. b-c) HRTEM images of 3% Pd-Nb_2O_5 interface. For the 3% sample, Pd particles are round with no obvious facets.

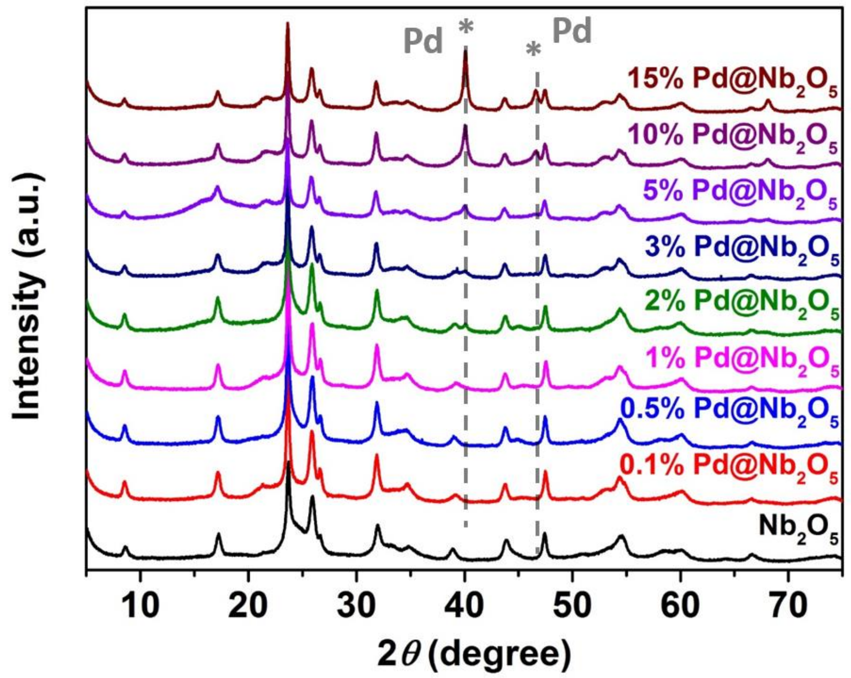

Figure S4. Powder X-ray diffraction patterns of Nb_2O_5, 0.1% Pd@Nb_2O_5, 0.5% Pd@Nb_2O_5, 1% Pd@Nb_2O_5, 2% Pd@Nb_2O_5, 3% Pd@Nb_2O_5, 5% Pd@Nb_2O_5, 10% Pd@Nb_2O_5 and 15% Pd@Nb_2O_5.

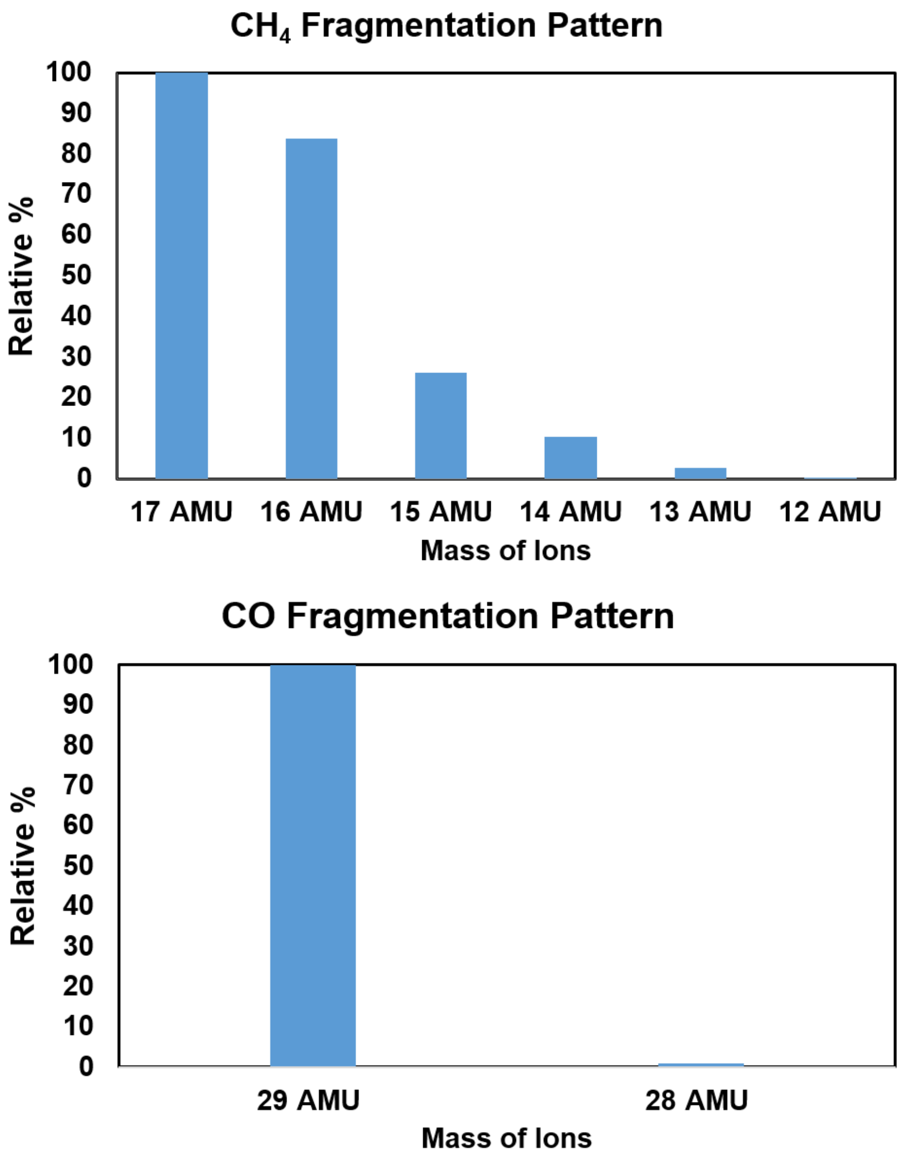

Figure S5. Fragmentation patterns measured by GC-MS for different products. On the top is CH_4 and on the bottom is CO.

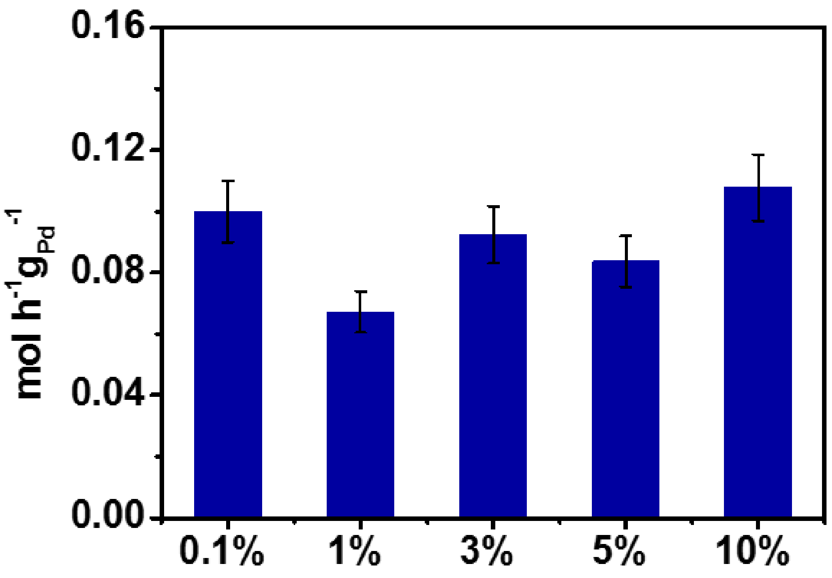

Figure S6. CH_4 production rates over $Pd@Nb_2O_5$ film samples with different Pd loadings under irradiation from a 300 W Xe lamp.

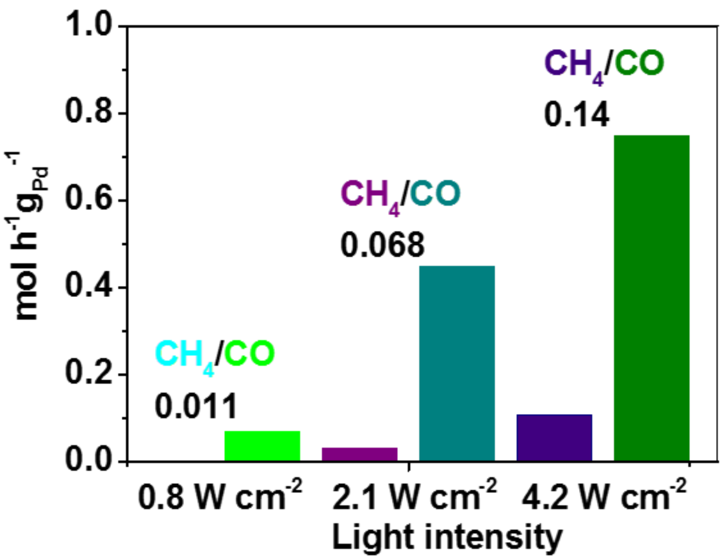

Figure S7. CO and CH_4 production rates over 10% $Pd@Nb_2O_5$ under different light intensities. The CH_4 selectivity is positively correlated with the illumination intensity.

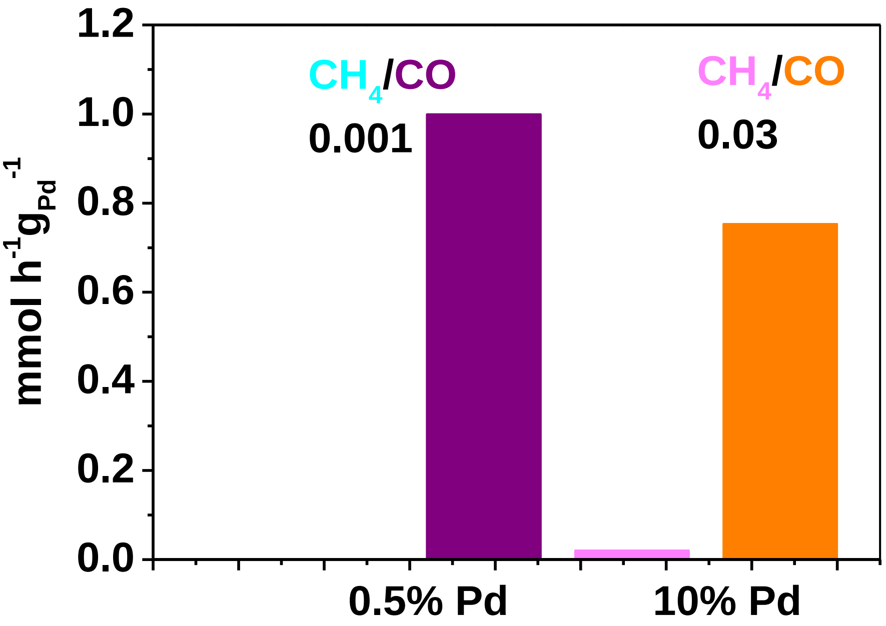

Figure S8. CO production rates, CH_4 production rates, and selectivity over 0.5% Pd@Nb_2O_5 and 10% Pd@Nb_2O_5 under 160°C in the dark.

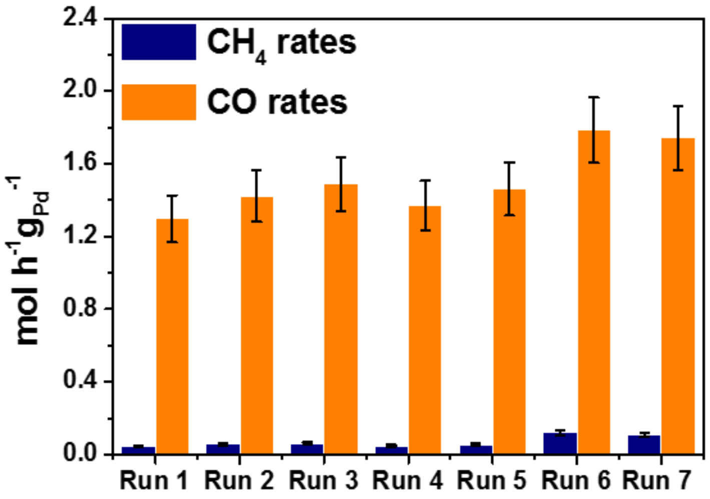

Figure S9. CO and CH_4 production stability on the presence of light for 3% Pd@Nb_2O_5 sample, where each test lasted for 30 minutes.

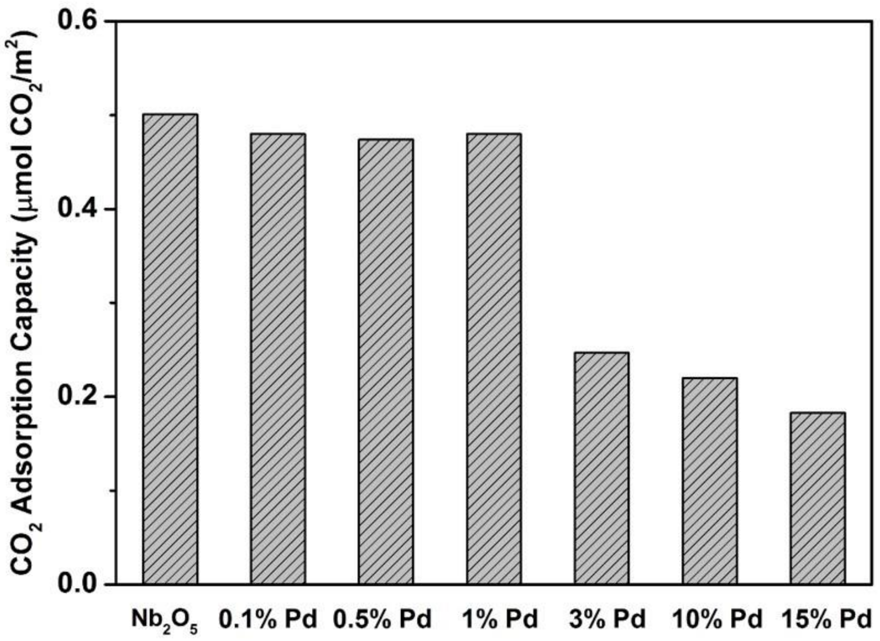

Figure S10. The dependence of the CO_2 adsorption capacity on the Pd loading in hybrids. The CO_2 adsorption capacity is normalized to the surface area of each sample.

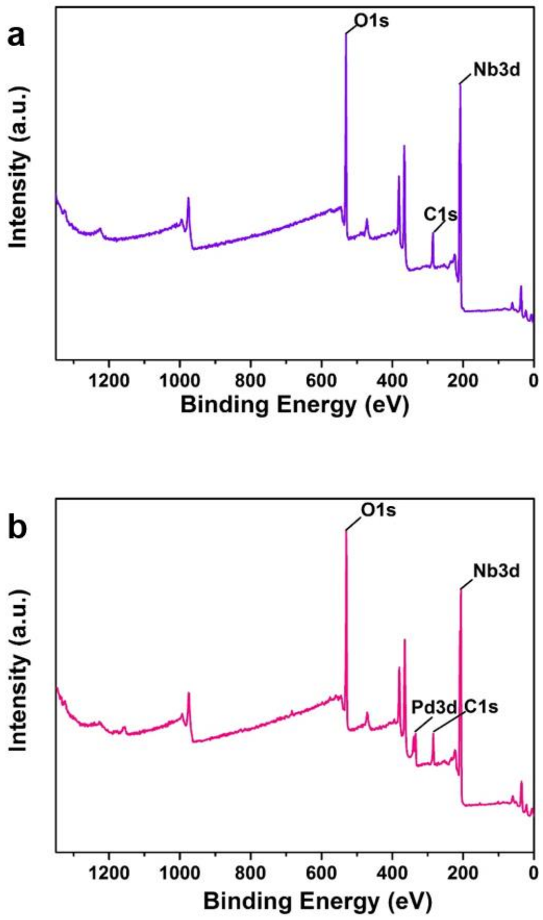

Figure S11. XPS spectra of the survey taken on a) Nb_2O_5 and b) 10% Pd@Nb_2O_5.

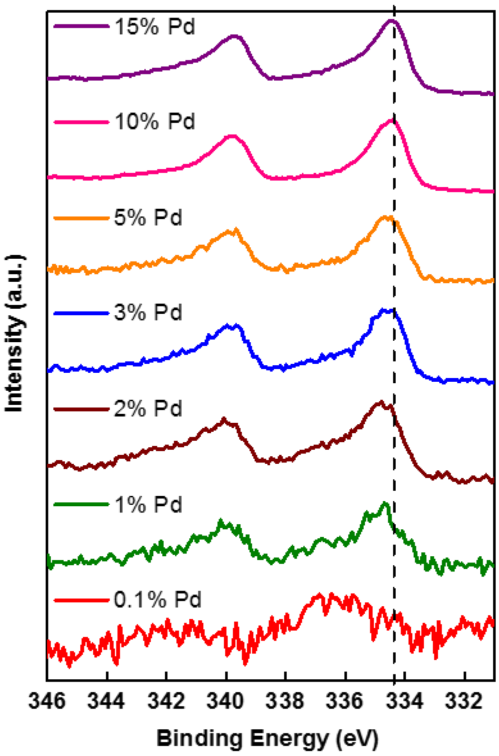

Figure S12. High resolution Pd 3d XPS spectra

	Pd(111)	Pd(211)	Pd55 nanoparticle
Top view	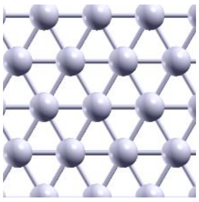	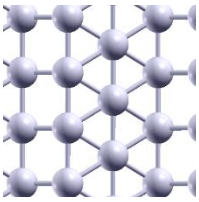	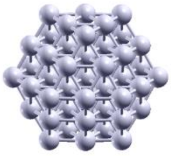
Side view	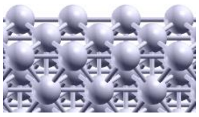	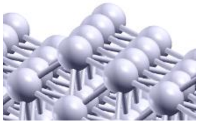	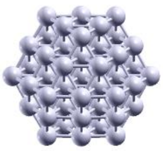

Figure S13. Atomic models used to simulate the Pd(111) and Pd(211) surfaces and Pd nanoparticle in DFT calculations.

	Initial state	Transition state	Final state
Pd(111)	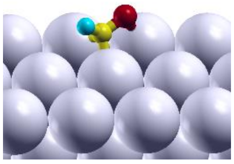	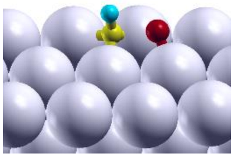	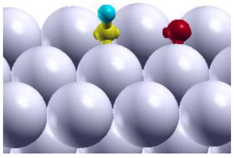
Pd(211)	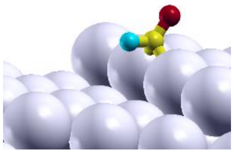	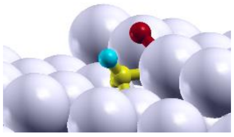	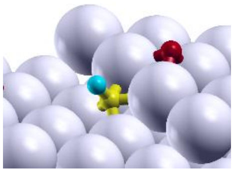
Pd55	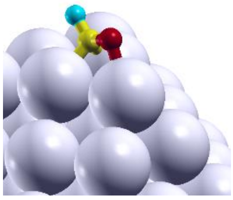	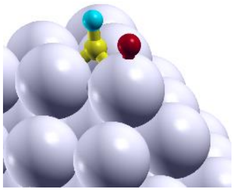	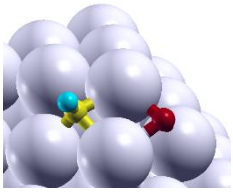

Figure S14. Atomic configurations of the initial, final, and transition states for C-O cleavage reactions of *CHO over Pd(111), Pd(211), and Pd55.

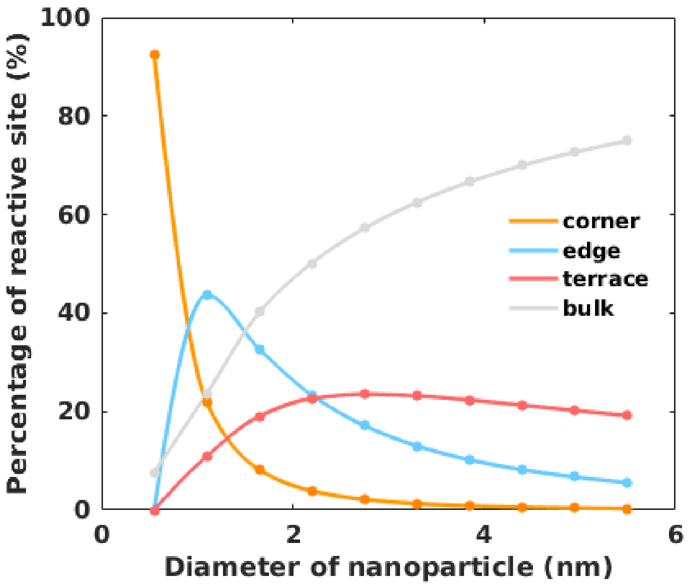

Figure S15. The weight fraction of adsorption sites (step, corner, terrace and bulk sites) on ideal octahedral Pd NPs as a function of the cluster diameter.

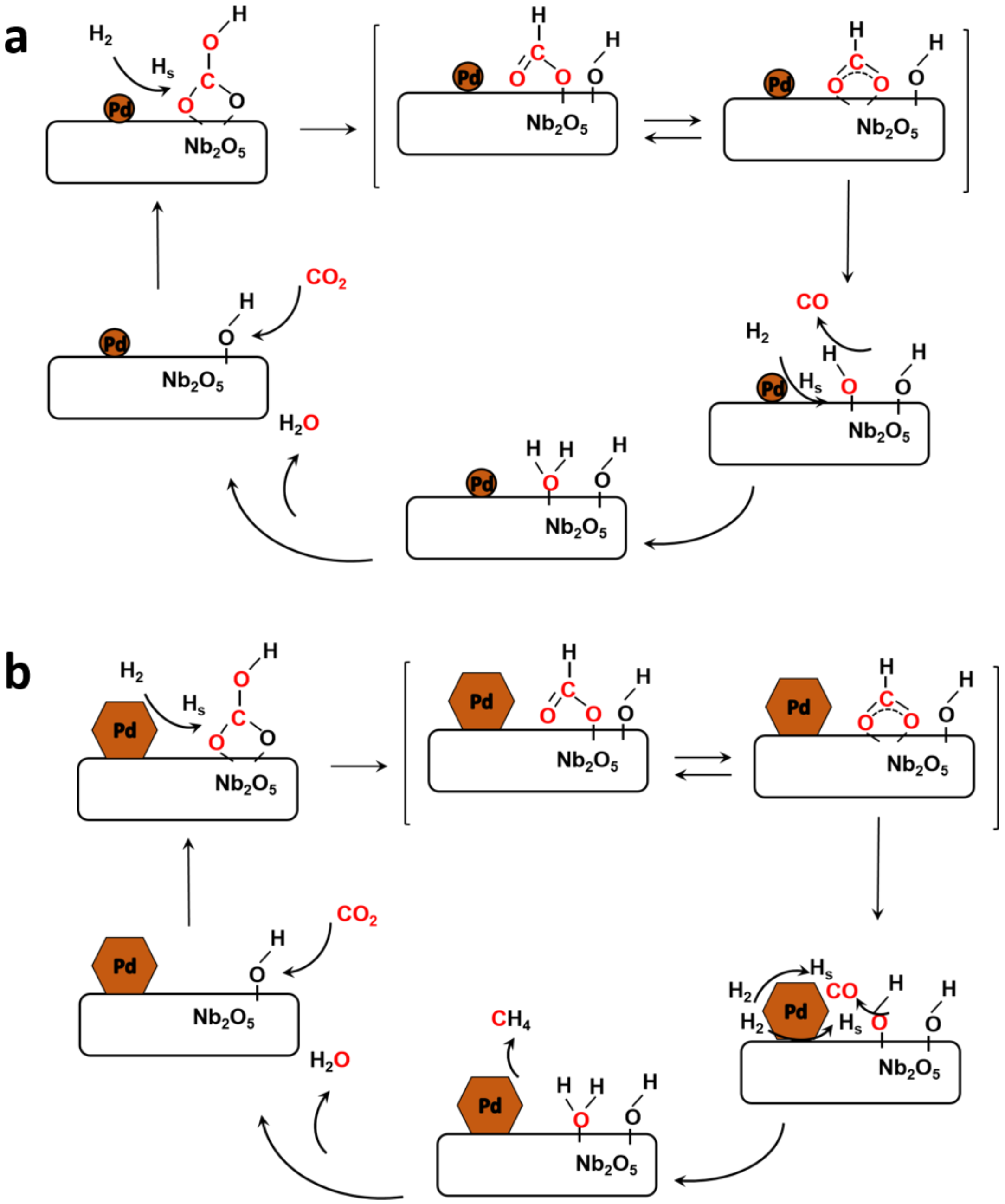

Figure S16. Reaction schemes for a) the reverse water gas shift reaction (RWGS) on a low Pd loading catalyst and b) the methanation reaction on a high Pd loading catalyst.

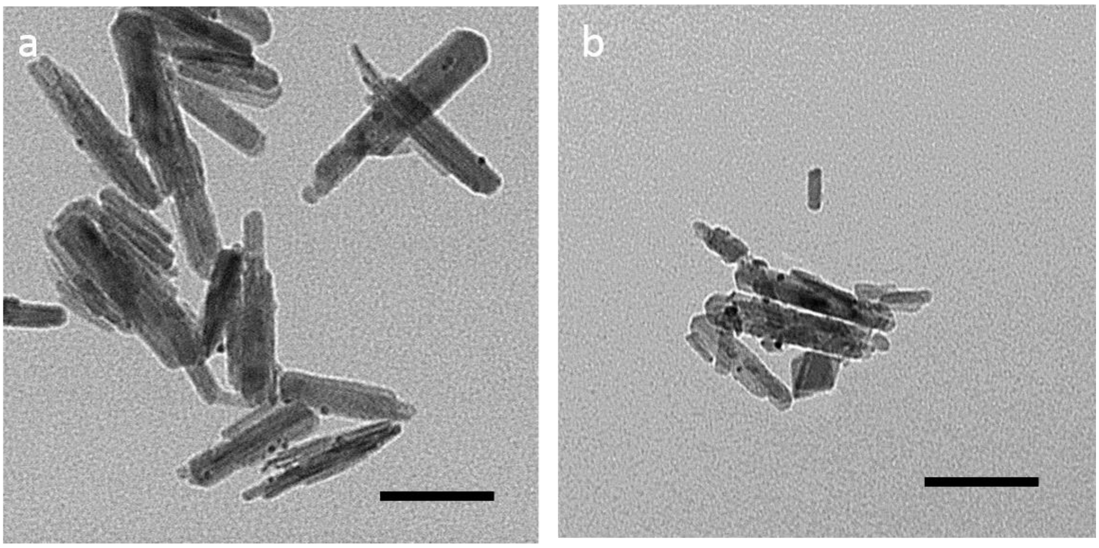

Figure S17. TEM images of 1%Pd@Nb_2O_5 a) before gas-phase catalytic testing, b) after 10 consecutive gas-phase catalytic tests, where each test lasted for 30 minutes. Scale bar: 100 nm.

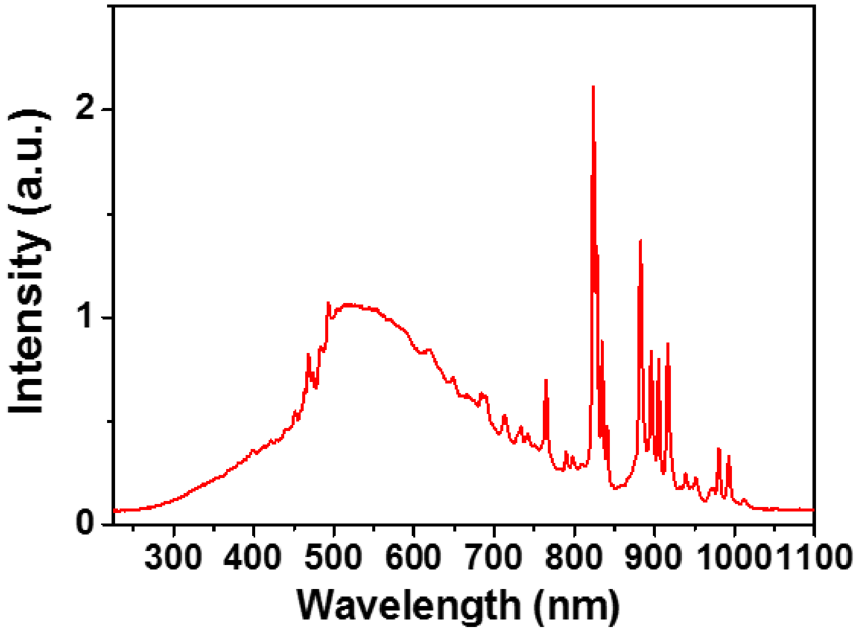

Figure S18. The emission spectrum of the 300 W Xe lamp.

Table S1. Elemental analysis of different Pd loading samples

Sample	Pd weight percentage
0.1% Pd@Nb_2O_5	0.14 wt%
0.5% Pd@Nb_2O_5	0.61 wt%
1% Pd@Nb_2O_5	1.13 wt%
2% Pd@Nb_2O_5	2.14 wt%
5% Pd@Nb_2O_5	5.64 wt%
10% Pd@Nb_2O_5	10.06 wt%

Table S2. Parameter values obtained from Pd K-edge EXAFS fitting. Values marked with an asterisk indicate that these parameters were correlated during EXAFS fitting.

**Sample**	**Path**	**CN**	**R / Å**	**σ^2 / 10^{-3} Å^2**	**ΔE_0 / eV**
Pd	Pd–Pd	10(1)	2.749(4)	2.9(6)	3(1)
PdO	Pd–O	3.7(4)	2.025(6)	1.9(9)	1.9(8)*
	Pd–Pd	4.5(7)	3.050(5)	3.3(8)	1.9(8)*
3% Pd	Pd–Pd	10(1)	2.751(4)	2.0(6)	4(1)
1% Pd	Pd–Pd	9(1)	2.752(4)	2.9(5)	4.1(9)
0.1% Pd	Pd–Pd	6.4(7)	2.746(4)	4.6(6)	3.3(9)

Table S3. The surface areas for the different Pd loading samples

Sample	Surface areas ($m^2 \cdot g^{-1}$)
Nb_2O_5	82
0.1% Pd@Nb_2O_5	81
0.5% Pd@Nb_2O_5	82
1% Pd@Nb_2O_5	83
2% Pd@Nb_2O_5	83
3% Pd@Nb_2O_5	80
5% Pd@Nb_2O_5	92
10% Pd@Nb_2O_5	89
15% Pd@Nb_2O_5	80

Table S4. CO chemisorption, the amount of exposed Pd surface atoms, apparent metal dispersion, CO production rates, CH_4 production rates, Nb_2O_5 active sites, and turnover frequency (TOF) for 3% Pd@Nb_2O_5 and 10% Pd@Nb_2O_5 under 4.2 W cm^{-2} in light-batch system and for 10% Pd@Nb_2O_5 under 2.1 W cm^{-2} in light-flow system.

	3% Pd@Nb_2O_5	10% Pd@Nb_2O_5	10% Pd@Nb_2O_5 (tested in flow reactor under 2.1 W cm^{-2})
CO chemisorption (μmol g^{-1})	26.36	41.71	41.71
The amount of exposed Pd surface atoms (μmol g^{-1})[a]	26.36	41.71	41.71
Apparent metal dispersion D (%)[a]	9.3%	4.4%	4.4%
CO production rate (mol h^{-1} g_{Pd}^{-1})	1.6	0.75	0.91
CH_4 production rate (mol h^{-1} g_{Pd}^{-1})	0.092	0.11	0.024
$TOF_{Pd, CO}$ (s^{-1})[c]	0.51	0.50	0.61
$TOF_{Pd, methane}$ (s^{-1})[d]	0.03	0.073	0.016
Nb_2O_5 active sites[e] (μmol g^{-1})	19.8	19.7	19.7
$TOF_{Nb2O5, CO}$ (s^{-1})[f]	0.69	1.05	1.29
$TOF_{Nb2O5, methane}$ (s^{-1})[g]	0.039	0.152	0.034

[a] The number of exposed Pd surface atoms is calculated from CO chemisorption using a Pd:CO = 1:1 stoichiometry ratio. The total number of Pd atoms is estimated using the loading amount of Pd. [c,d] We calculate the TOF_{Pd} numbers based on the exposed Pd surface atoms. [e] The number of Nb_2O_5 active sites is calculated by multiplying the CO_2 capture capacity by the surface area of each sample. [f,g] The TOF_{Nb2O5} numbers are determined based on the Nb_2O_5 active sites.

Table S5. The energy conversion efficiency in light-batch system over 0.1% Pd@Nb_2O_5 under 4.2 W cm^{-2} and in light-flow system over 10% Pd@Nb_2O_5 under 2.1 W cm^{-2}.

	0.1% Pd@Nb_2O_5 (Batch)	10% Pd@Nb_2O_5 (Flow)
CO production rate (mol $h^{-1}g_{Pd}^{-1}$)	18.8	0.70
Sample mass of Pd@Nb_2O_5 (mg)	2.5	5.6
$CO_2(g) + H_2(g) \rightleftharpoons CO(g) + H_2O(g)$ $\Delta H^{\circ}_{298.15\ K}$ (kJ mol^{-1})	41.17	41.17
The change in enthalpy[a]	0.000537 W h	0.00449 W h
Light energy[b]	4.2 W h	2.1 W h
Absorbed light energy[c]	1.18 W h	1.97 W h
Energy conversion efficiency[d]	0.05%	0.23%

[a] The change in enthalpy is estimated as the the product of the CO production rate, the sample mass, and $\Delta H^{\circ}_{298.15\ K}$ for the RWGS reaction. for the RWGS reaction. [b] The light energy is calculated by multiplying the incident light intensity (4.2 W cm^{-2} or 2.1 W cm^{-2}) by the illumination area of the film (1 cm^2). The incident spectrum for photons with energy greater than 1.13 eV is shown in **Fig. S18**. [c] The absorbed light energy is calculated by multiplying the incident light by the absorption of the catalyst for each photon wavelength, and then summing over the incident spectrum. [d] The energy conversion efficiency is reported as the increased enthalpy of the system after the reaction divided by the absorbed light energy. The energy conversion efficiency compares the light energy absorbed by the catalyst to the increase in enthalpy of the system, however it should be noted that the change in entropy of the system is not taken into consideration.

Table S6. Locations of surface sites used in DFT calculations

Site	Pd(111)	Pd(211)	Pd55
	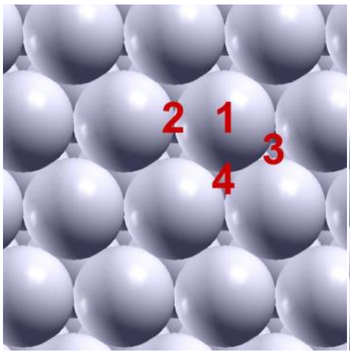	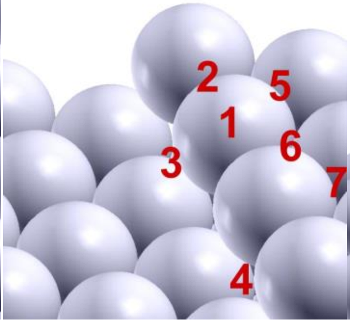	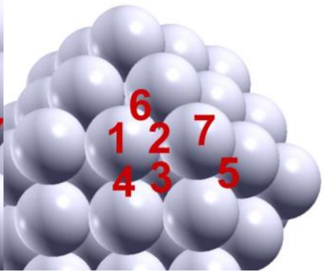
1	Top	Top@211-edge	Top@211-edge
2	Bridge	Bridge@211-edge	Bridge@211-edge
3	Face-centred-cubic (fcc)	Bridge@100-edge	Hollow@100-edge
4	Hexagonal-close-packing (hcp)	Hollow@100-edge	Bridge@100-edge
5	-	Bridge@111-edge	Hollow@111-edge
6	-	Hollow@111-edge (1)	Bridge@111-edge
7	-	Hollow@111-edge (2)	Top@corner

Table S7. Adsorption energies of relevant intermediates on different reactive sites. Corresponding site locations are shown in in **Table S6**. Values marked in bold denote the adsorption energy of the most stable intermediate. Note that for the *CO, *CHO, and *CH intermediates on Pd(111), only the fcc site has been calculated since previous studies have shown fcc to be the most stable adsorption site in all three of these cases. [1]

Site		1	2	3	4	5	6	7
Pd(111)	*CO note			**-1.954**				
	*CHO note			**-2.266**				
	*CH note			**-6.815**				
	*CH2	-2.755	-3.771	-3.770	**-3.776**			
	*CH3	**-1.723**	unstable	-1.560	-1.481			
	*O	unstable	unstable	**-4.433**	-4.261			
Pd(211)	*CO	-1.505	-1.888	-1.593	-1.893	unstable	**-1.920**	-1.825
	*CHO	-2.314	**-2.419**	unstable	-2.154	unstable	-2.332	unstable
	*CH	unstable	unstable	unstable	**-6.716**	unstable	-6.685	-6.597
	*CH2	unstable	**-3.986**	-3.661	unstable	-3.647	unstable	unstable
	*CH3	-1.801	**-1.853**	unstable	unstable	unstable	unstable	unstable
	*O	unstable	-4.248	unstable	-3.982	unstable	**-4.315**	-4.301
Pd55	*CO	-1.586	-1.938	-1.795	-1.910	**-2.051**	unstable	-1.621
	*CHO	-2.422	-2.401	unstable	-2.388	unstable	**-2.503**	-2.223
	*CH	unstable	unstable	**-6.953**	-5.886	-6.670	unstable	-4.174
	*CH2	-2.741	**-3.891**	unstable	-3.847	-3.669	-3.614	-2.949
	*CH3	-1.822	unstable	unstable	unstable	unstable	-1.544	**-1.900**
	*O	-3.272	-4.248	unstable	-4.092	**-4.487**	unstable	unstable

[1] a) S. Lin, J. Ma, X. Ye, D. Xie, H. Guo, *J. Phys. Chem. C* **2013**, 117, 14667; b) R. Zhang, H. Liu, B. Wang, L. Ling, *J. Phys. Chem. C* **2012**, 116, 22266.
